# Rapid and Reusable Amino Acid–Derived MOFs for the Removal of Estrogenic Hormones From Real Water Matrices

**DOI:** 10.1002/chem.71554

**Published:** 2026-08-13

**Authors:** Lidia García, Alejandro Cortés‐Villena, Héctor Martínez Pérez‐Cejuela, José Manuel Herrero‐Martínez, Jesús Ferrando‐Soria, Thais Grancha, Donatella Armentano, Emilio Pardo

**Affiliations:** ^1^ Instituto de Ciencia Molecular (ICMol) Universidad de Valencia Valencia Spain; ^2^ Department of Analytical Chemistry University of Valencia Valencia Spain; ^3^ Dipartimento di Chimica e Tecnologie Chimiche (CTC) Università della Calabria Cosenza Italy

**Keywords:** endocrine‐disrupting compounds, estrogen removal, metal–organic frameworks, regenerable adsorbents, solid‐phase extraction (SPE), water remediation

## Abstract

Estrogenic hormones are potent endocrine‐disrupting contaminants that are inefficiently removed by conventional wastewater treatment processes. Herein, we evaluate a family of isoreticular amino‐acid–derived metal–organic frameworks (MOFs), including multivariate variants, for the simultaneous capture of estrone (E1), 17β‐estradiol (E2), estriol (E3), and 17α‐ethinylestradiol (EE2) from water. Using a solid‐phase extraction configuration, a leucine‐based oxamidato MOF emerges as a highly efficient sorbent, achieving >90%–95% removal of all four hormones within 30 s in a single loading step, representing one of the fastest simultaneous estrogen capture systems reported to date under SPE conditions. This material exhibits exceptional regenerability, enabling near‐quantitative desorption under mild conditions with minimal solvent consumption (1 mL methanol), and stable performance over at least ten reuse cycles. Notably, comparable capture efficiencies are maintained in real river‐water samples, demonstrating robustness toward complex aqueous matrices. The crystal structure of a host–guest aggregate confirms estrogen encapsulation within the flexible framework, providing direct structural insight into the origin of the high capture efficiency. These results identify amino‐acid–based MOFs as a highly competitive platform for rapid, regenerable, and selective estrogen capture, combining excellent performance with valuable structural insight into host–guest interactions.

## Graphical abstract

A family of amino‐acid–derived MOFs enables ultrafast (>90–95% in 30 s) simultaneous removal of four estrogenic contaminants from water under SPE conditions. A leucine‐based MOF shows excellent regenerability and robustness in real samples, with structural evidence revealing efficient hormone encapsulation.

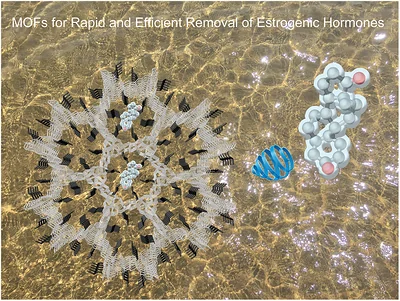

## 1 Introduction

Natural estrogens such as estrone (E1), 17β‐estradiol (E2), and estriol (E3), along with the synthetic estrogen 17α‐ethinylestradiol (EE2) commonly used in oral contraceptives (Scheme S1), are among the most potent endocrine‐disrupting compounds (EDCs) [1] identified in aquatic environments [2]. These contaminants enter water systems primarily through wastewater discharges, agricultural runoff, and pharmaceutical residues [2]. Even at trace concentrations, they can disrupt the hormonal balance of aquatic organisms, leading to feminization of male fish, intersex conditions, impaired reproduction, and population‐level declines [3]. Their persistence and strong biological activity pose serious environmental risks, particularly since conventional wastewater treatment plants (WWTPs) often fail to remove them efficiently [4, 5, 6]. The frequent detection of estrogens in surface waters, therefore highlights the urgent need for more efficient, selective, and sustainable remediation strategies [7].

Numerous treatment technologies have been explored for estrogen removal, each with distinct advantages and drawbacks. Conventional biological treatments (e.g., activated sludge, constructed wetlands) often exhibit limited removal efficiencies due to the low concentrations and recalcitrant nature of these compounds [8]. Advanced oxidation processes, such as ozonation and UV/H_2_O_2_, can achieve degradation through radical pathways, but are energy‐intensive and may produce toxic byproducts [9]. Activated carbon adsorption, though widely employed for micropollutant removal, suffers from limited regeneration [10] and progressive saturation [11], which is particularly evident in the presence of other competitive adsorbents [12]. Membrane‐based processes [13] (nanofiltration, reverse osmosis) achieve high rejection rates but are hindered by fouling, high energy demand, and operational costs [13]. Emerging adsorbents such as molecularly imprinted polymers (MIPs) and biochar show enhanced selectivity, yet their scalability and long‐term stability remain under evaluation. Overall, developing cost‐effective, regenerable, and environmentally benign materials for efficient estrogen capture remains a major challenge [14].

Metal–organic frameworks [15, 16, 17] (MOFs) have recently emerged as highly promising candidates for the adsorption and removal of estrogens from water [18]. These crystalline hybrid materials –constructed from metal nodes and organic linkers– combine exceptionally high surface areas, tunable pore structures, and versatile chemical functionality [19]. Such features enable MOFs to interact selectively with trace organic pollutants through hydrogen bonding, π–π stacking, hydrophobic effects, and electrostatic interactions. In contrast to conventional adsorbents such as activated carbons, MOFs can be easily rationally designed to enhance affinity toward specific molecules by modifying their pore environment and surface chemistry. Furthermore, their structural robustness allows integration into membranes [20], foams [21], or composite systems [21] to improve stability and reusability during multiple adsorption–desorption cycles.

Several MOFs have been explored for the removal of estrogenic contaminants from water, showing promising but still limited performance [22]. For instance, UiO‐66(Zr) [23], amino‐functionalized MIL‐101 [24, 25] and MIL‐53(Fe) [26] and related Fe_3_O_4_@MIL‐101 [27] composites have been applied successfully as rapid sorbents for analytical solid‐phase extraction (SPE)/preconcentration of estrogens (E1, E2, E3 and EE2 separately), achieving good recoveries in spiked waters (≈80%–100%) at µg L^−^^1^ levels. Cerium‐doped DUT‐52 has similarly been developed as a dispersive SPE sorbent for estrogens in biological matrices, showing high recoveries and moderate cycle‐to‐cycle reusability under analytical dispersive SPE conditions [28]. Calcium‐based MOFs (e.g., Ca‐MIX) have also been investigated in batch adsorption studies for EE2 and related hormones, demonstrating measurable uptake and sensitivity to pH, ionic strength, and natural organic matter; however, reported capacities are generally µg·g^−^^1^ scale, with equilibrium times of several hours [29].

Despite these advances, the practical application of MOFs for estrogen remediation still faces several challenges. In particular, no single system has yet achieved the simultaneous combination of ultrafast uptake, efficient capture of multiple hormones, facile regeneration with minimal solvent use, and validated performance in real water matrices [22, 23, 24, 25, 26, 27, 28, 29].

Most systems operate under batch conditions, requiring relatively long contact times to reach equilibrium, which limits their use in continuous water‐treatment setups [30]. Regeneration often relies on large volumes of organic solvents or elevated temperatures, raising sustainability and cost concerns. Moreover, the stability and recyclability of many MOFs in real aqueous environments remain insufficiently established [31, 32], as repeated exposure to water, natural organic matter, or varying ionic strength can induce structural degradation or reduce adsorption performance. Finally, comprehensive evaluations in realistic matrices, such as surface or wastewater samples containing multiple hormones and coexisting organic matter and ions, are still scarce. Addressing these issues requires the development of robust, rapidly acting, and easily regenerable MOFs capable of selective multi‐hormone capture under continuous‐flow and environmentally relevant conditions.

In this work, we aim to address these limitations by developing a system capable of simultaneous, ultrafast, and regenerable capture of multiple estrogenic hormones (E1, E2, E3 and EE2) under flow‐like SPE conditions, while maintaining performance in environmentally relevant matrices. To this end, we propose using a family of isoreticular, amino‐acid–derived MOFs, as well as multivariate (MTV)‐MOFs, which have previously proven highly effective for the removal of emerging contaminants such as heavy metals [33, 34, 35, 36, 37], insecticides [38], organic dyes [39, 40], antibiotics [41], pharmaceuticals and illicit drugs [30], and even the highly concerning PFAS [42, 43]. These MOFs share the same topology but incorporate different side chains depending on the amino acids decorating their medium‐sized hexagonal channels. This strategy will provide an initial platform for identifying the MOF with the most suitable functionalities for hormone capture. Subsequently, other key parameters of the selected material can be evaluated, including its regeneration capacity and its performance over successive reuse cycles.

In particular, the selected battery of MOFs and MTV‐MOFs is four oxamidato‐based MOFs with formulas Ca^II^Cu^II^_6_[(*S,S*)‐alamox]_3_(OH)_2_(H_2_O)} ^.^ 32H_2_O [44] (**1**), {Ca^II^Cu^II^_6_[(*S,S*)‐methox]_3_(OH)_2_(H_2_O)} ^.^ 16H_2_O [33, 34] (**2**) and {Ca^II^Cu^II^_6_[(*S*,*S*)‐leumox]_3_(OH)_2_(H_2_O)}·11H_2_O [45] (**3**) and {Sr^II^Cu^II^_6_[(*S*,*S*)‐threonine]_3_(OH)_2_(H_2_O)}·36H_2_O [46] (**4**), as well as two multivariate (MTV)‐MOFs –incorporating two different amino acid–derived ligands within the same structure– with formulas {Ca^II^Cu^II^_6_[(*S,S*)‐methox]_1.5_[(*S,S*)‐serimox]_1.50_(OH)_2_(H_2_O)} ^.^ 30H_2_O [47] (**5**) and {Sr^II^Cu^II^_6_[(*S,S*)‐methox]_1.5_[(*S,S*)‐Mecysmox]_1.50_(OH)_2_(H_2_O)} ^.^ 36H_2_O [38, 48] (**6**) (Figure 1). The oxamidate ligands are derived from their corresponding amino acids and are denoted as follows: alamox = bis[(S)‐alanine]oxalyl diamide; serimox = bis[(S)‐serine]oxalyl diamide; methox = bis[(S)‐methionine]oxalyl diamide; threomox = bis[(S)‐threonine]oxalyl diamide; leumox = bis[(S)‐leucine]oxalyl diamide; and Mecysmox = bis[(S)‐methylcysteine]oxalyl diamide (Scheme S2).

**FIGURE 1:**
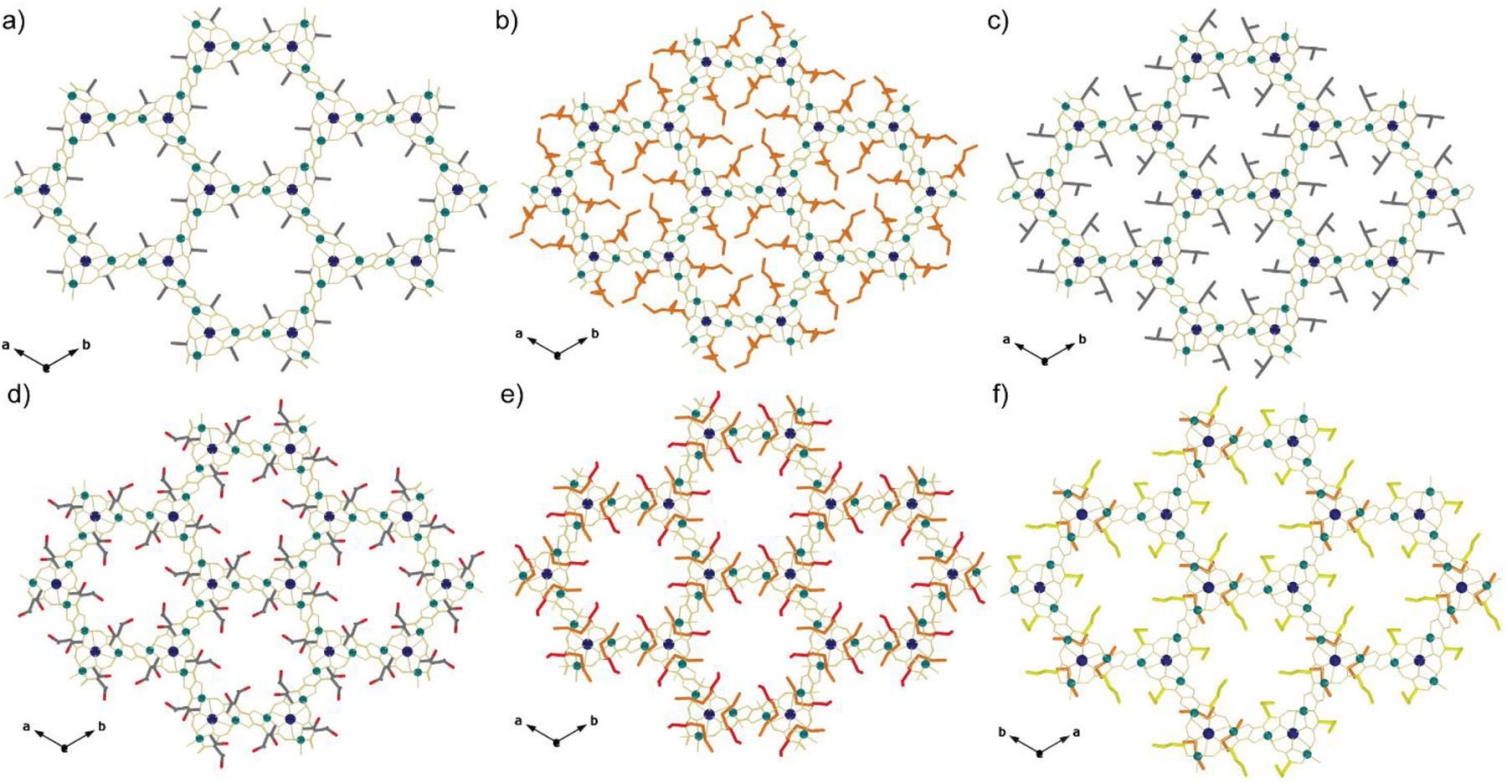
Structural representation of MOFs **1**–**4** (a–d) and MTV‐MOFs **5** (e) and **6** (f). Metal centers are depicted as blue spheres for Ca^II^/Sr^II^ and cyan spheres for Cu^II^, while the organic linkers are shown as gold sticks. Amino acid side chains are highlighted using thicker representations and color‐coded according to their chemical functionality: red (─CH_2_OH, *L*‐serine), yellow (─CH_2_SCH_3_, *S*‐methyl‐*L*‐cysteine), orange (─CH_2_CH_2_SCH_3_, *L*‐methionine), gray (─CH_3_, *L*‐alanine; ─CH_2_CH(CH_3_)_2_, *L*‐leucine), and red/gray (─CH(OH)CH_3_, *L*‐threonine).

## 2 Results and Discussion

MOFs and MTV‐MOFs **1**–**6** were prepared according to previously reported procedures (see Experimental Section) [33, 34, 38, 44, 45, 46, 47, 48].

Prior to evaluating their adsorption performance, the structural integrity and porosity of the materials were assessed by powder X‐ray diffraction (PXRD) and nitrogen adsorption measurements. The diffraction patterns were consistent with the expected crystalline phases, confirming successful synthesis of the targeted frameworks (Figures S1 and S2). Nitrogen adsorption isotherms further evidenced the presence of permanent porosity, displaying profiles in agreement with those previously described for these materials (Figure S3). The Brunauer–Emmett–Teller (BET) surface areas [49] were determined to be 1029.2, 164.7, 357.9, 706.8, 637.9, and 553.5 m^2^ g^−^^1^ for compounds **1**–**6**, respectively.

The crystal structures of compounds **1**–**6** are illustrated in Figure 1. All materials belong to an isoreticular series and crystallize in the chiral *P*6_3_ space group within the hexagonal crystal system. Their architectures consist of 3D networks built from Ca(II) or Sr(II) nodes interconnected by Cu(II) centers, giving rise to hexagonally arranged channels with pore apertures in the 0.3–0.8 nm range. Within these channels, amino acid–derived side chains are oriented toward the empty pore space, providing a chemically diverse and conformationally flexible environment. Such flexibility allows the frameworks to accommodate guest molecules of different sizes and shapes through adaptive structural responses. In the multivariate analogues (**5** and **6**), the simultaneous incorporation of two distinct amino acid functionalities further increases the chemical heterogeneity of the pore Surface (Figure 1e,f), which may influence guest–host interactions. Altogether, the combination of structural robustness and internal adaptability makes this family of MOFs suitable for interactions with a wide variety of organic molecules.

The adsorption performance of MOFs **1**–**6** toward a mixture of estrogenic hormones –estrone (E1), 17β‐estradiol (E2), estriol (E3), and 17α‐ethinylestradiol (EE2)– was initially evaluated under solid‐phase extraction (SPE) conditions. For this purpose, aqueous solutions (pH 7.0) containing all four compounds at a concentration of 2 mg L^−1^ each were processed through SPE cartridges packed with the corresponding materials. The cartridges were prepared by loading 50 mg of each MOF between two porous frits inside 1 mL polypropylene columns. After percolation, the collected fractions were passed through 0.22 µm PTFE filters and analyzed by high‐performance liquid chromatography (HPLC) to determine the residual hormone concentrations (see Experimental Section and Tables S1–S4). Although estrogens are commonly detected in natural waters at ng–µg L^−1^ concentrations, the primary objective of this work is not to reproduce environmental levels but to evaluate and compare the intrinsic adsorption performance of a family of structurally related MOFs under controlled SPE conditions. Accordingly, a concentration of 2 mg L^−^^1^ was selected to ensure reliable quantitative analysis by HPLC–fluorescence while maintaining sufficient discrimination between materials. Although this concentration exceeds those typically encountered in environmental waters, it provides a robust proof‐of‐concept assessment of the adsorption performance under SPE conditions and facilitates comparison among the different MOFs.

Overall, two MOFs (**3** and **5**) displayed superior performance in this screening (Figure 2), whereas MOFs **1**, **2**, **4**, and **6** exhibited more modest capture capacities, far from benchmark adsorbents such as activated carbons [11]. In particular, MOF **3** showed excellent capture properties, achieving simultaneous removal of E1, E2, E3, and EE2 very efficiently (92%, 93%, 96%, and 94%, respectively) in a single loading step within 30 s (Figure 2a and Table S1). Even better capture results were obtained for MTV‐MOF **5** (96%, 98%, 99%, and 98%, respectively), likely arising from cooperative effects between the two different functional groups (─CH_2_SCH_3_ and ─CH_2_CH_2_SCH_3_) decorating its channels. These results approach the performance of benchmark adsorbents such as activated carbons in terms of removal efficiency [11].

**FIGURE 2:**
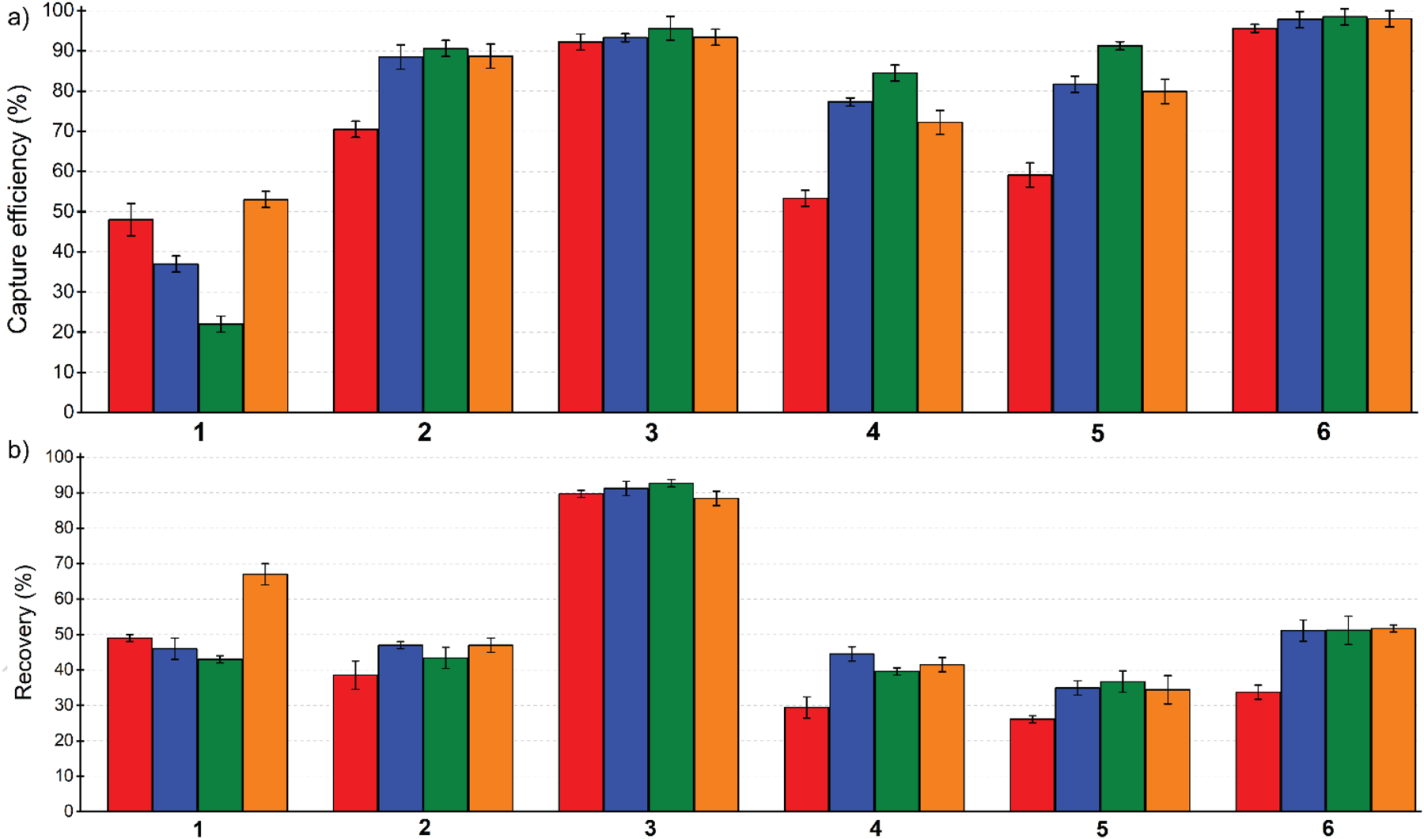
(a) Removal efficiency (%) of E1 (red), E2 (blue), E3 (green), and EE2 (orange) from aqueous solutions (2 mg L^−^^1^ each) using MOFs **1**–**6**. (b) Desorption efficiency (%) of the same compounds after elution with 1 mL of methanol. Error bars correspond to three independent experiments.

The regeneration behavior of MOFs **1**–**6** was subsequently evaluated following the adsorption experiments. To this end, 1 mL of CH_3_OH was percolated through the cartridges containing the corresponding MOF, and the eluates were analyzed (see Figure 2b and Table S2). Interestingly, MOFs **1**, **2**, and **4**–**6** showed a rather modest recovery, with more than 50% of the estrogens remaining retained in the respective MOFs. Nevertheless, this represents an improvement compared to activated carbon, which showed no recovery at all [11]. In contrast, MOF **3** exhibited an exceptional recovery, with practically 100% of the estrogens retained being later eluted with just 1 mL of methanol in 15 s (Figure 2b). The distinct behavior of MOFs **3** and **5** suggests a trade‐off between adsorption strength and reversibility, in which enhanced functional diversity promotes higher capture efficiency but compromises desorption, which is crucial for reusability cycles and cost‐efficiency.

Given the high regeneration efficiency observed for MOF **3**, additional experiments were conducted to assess its practical applicability under repeated use. First, we explored the reusability of MOF **3** against these four estrogens by determining the material's capture efficiency (%) after 10 consecutive cycles (taking measurements after 1, 3, 5, and 10 reuses). Thus, it can be observed that, at least over 10 capture cycles, no decrease in this efficiency is detected (Figure 3a and Table S3), remaining similar to that observed in Figure 2. This excellent reusability is most likely related to the high regeneration observed (Figure 2b). Note that, between each reuse, 2 mL of methanol and 2 mL of nanopure water are percolated through the cartridge, regenerating it (Figure 3a) and allowing a longer lifetime device. In addition, the integrity of MOF **3** after the 10^th^ capture experiment was confirmed by means of its PXRD pattern (Figure S4), which is identical to that observed for the fresh polycrystalline sample.

**FIGURE 3:**
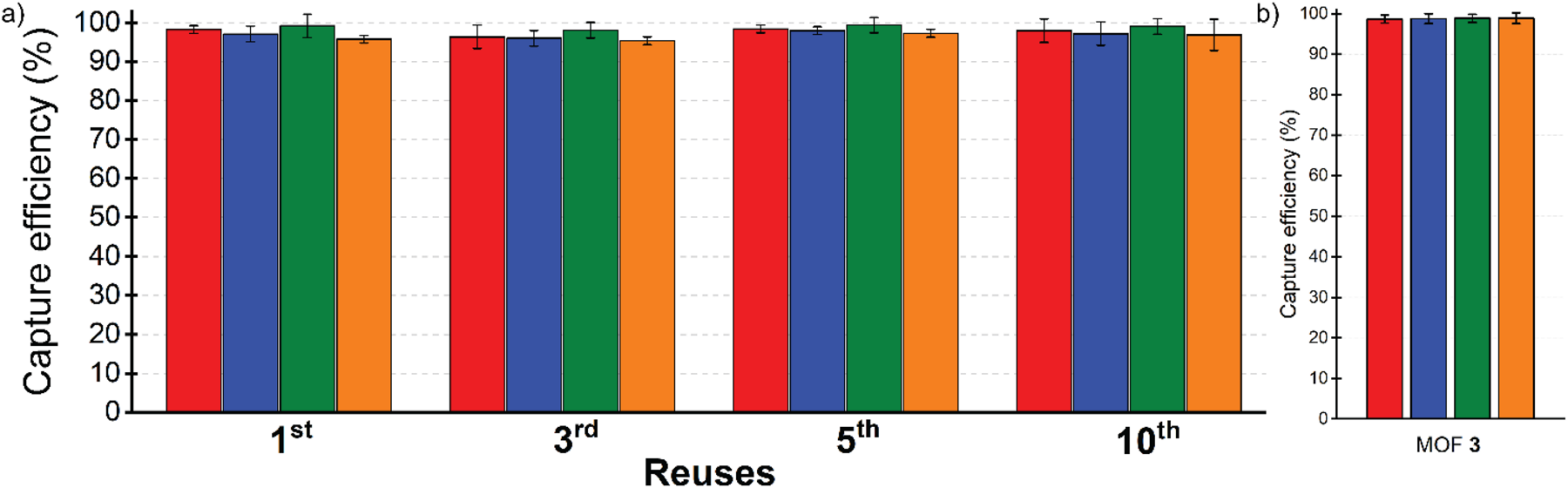
(a) Reuses of MOF **3** for the removal (%) of E1 (red), E2 (blue), E3 (green) and EE2 (orange) from an aqueous solution containing 2 mg L^−1^ of each pollutant. (b) Capture efficiency (%) of MOF **3** for E1 (red), E2 (blue), E3 (green) and EE2 (orange) from a Turia river water sample spiked with 2 mg L^−1^ of each estrogen. Error bars of three independent replicate measurements are included.

To further evaluate the long‐term stability of MOF **3**, additional characterization was performed after ten consecutive adsorption–desorption cycles. ICP–MS analyses of the collected solutions revealed no detectable Ca or Cu leaching, confirming the excellent chemical stability of the framework under the investigated SPE conditions. Moreover, the N_2_ adsorption isotherm and BET surface area (371 m^2^ g^−1^) of the recycled material were essentially identical to those of the pristine sample (Figure S5), demonstrating that the permanent porosity of the framework is fully preserved after repeated use. Together with the unchanged PXRD pattern (Figure S4), these results provide strong evidence for the outstanding robustness of MOF **3**.

Following the promising results obtained under controlled laboratory conditions, the performance of MOF **3** was further evaluated using a real water matrix. To this end, a sample collected from the Turia river (Valencia, 39.484965°N, −0.421584°W) was spiked with E1, E2, E3, and EE2 at concentrations of 2 mg L^−^^1^ each and subjected to the same SPE protocol described above.

MOF **3** maintained removal efficiencies above 98% for all four estrogens in the real water sample, values comparable to those obtained in ultrapure water (Figure 3b and Table S4). These results indicate that the presence of dissolved ions and natural organic matter does not significantly hinder the adsorption process. The slightly enhanced performance observed in the river‐water matrix may be associated with ionic strength effects, as previously reported for related systems [38, 39, 41]. This combination of framework stability and internal flexibility underpins the ability of these materials to interact with a broad range of organic guests.

Finally, to further benchmark the performance of MOF **3** against previously reported adsorbents, we evaluated its maximum uptake capacity toward each of the selected estrogens. Polycrystalline samples of **3** were immersed in saturated water/methanol (5:1, v/v) solutions of E1, E2, E3, and EE2 for five days, replacing the saturated solution every 12 h to ensure continuous exposure to excess guest molecules. Under these conditions, uptake capacities of 323, 429, 299, and 377 mg g^−^^1^ were determined for E1, E2, E3, and EE2, respectively (see Table S5 and Experimental Section in Supporting Information for further details). These values are fully comparable to those reported for the best‐performing estrogen adsorbents described to date (Table 1). However, adsorption capacity alone does not provide a complete assessment of adsorbent performance. As summarized in Table 1, MOF **3** additionally combines ultrafast adsorption kinetics, excellent regenerability with minimal solvent consumption, outstanding reusability, and validated performance in real water samples, features that are rarely achieved simultaneously by previously reported materials.

**TABLE 1:** Comparison of representative MOF‐ and non‐MOF‐based adsorbents reported for estrogen removal, highlighting the performance of the present work.

Material	Adsorption capacity	Kinetics	Regeneration efficiency	Real‐water performance	Operation conditions	Reference
Activated carbon	EE2 (3.16–7.10 mg /g, 96.4‐99.3%)	NR	NR	Waste water final effluent	0.1‐3.0 g 1L spiked solution (3 h, 3 distinct carbons from coal, coconut and wood)	12
Graphene nanomaterials	E2 (K_F_ = 64.39, 77.86) EE2 (K_F_ = 38.68, 61.41)	NR	NR	Simulated natural organic matter	1 mg (24 h)	11
Membrane‐based processes	E1 (91–98 %) E2 (92–98 %) E3 (83‐94 %) EE2 (92–94 %)	NR	NR	Modelled waters	Solid‐Phase Extraction [3 h at 2.0 MPa]	13
UiO‐66(Zr)	E1 (97.3, 98.7 and 99.5 % for 5, 50, and 100 ppb) E2 (96.7, 99.2 and 99.1 % for 5, 50, and 100 ppb)	NR	Four cycles	River water	Dispersive solid‐phase extraction	23
MIL‐101	E2 (77.76 mg/g) E3 (115.31 mg/g) EE2 (93.41 mg/g)	quasi‐second‐order	NR	NR	Batch adsorption [5 mg, EtOH solutions]	24
NH_2_‐MIL‐101	E2 (89.48 mg/g) E3 (117.72 mg/g)) EE2 (117.10 mg/g)	quasi‐second‐order	NR	NR	Batch adsorption [5 mg, EtOH solutions]	24
Br‐MIL‐101	E2 (82.01 mg/g) E3 (109.46 mg/g) EE2 (103.91 mg/g)	quasi‐second‐order	NR	NR	Batch adsorption [5 mg, EtOH solutions]	24
NO_2_‐MIL‐101	E2 (73.66 mg/g) E3 (108.73 mg/g)) EE2 (100.91 mg/g)	quasi‐second‐order	NR	NR	Batch adsorption [5 mg, EtOH solutions]	24
GMA‐NH_2_‐MIL‐101(Al)	E1 (90–92 % 25 ppb) E2 (79–86 % 5 ppb; 85–94 % 25 ppb) E3 (72–83 % 5 ppb; 73–79 % 25 ppb) EE2 (87‐89 % 5 ppb; 90‐94 % 25 ppb)	NR	NR	Tap water, river water and human urine	Stir‐bar sorptive extraction [5 ppb, 25 ppb each, 30 min 500 rpm]	25
MIL‐53(Fe)	EE2 (1.26 mg/g)	NR	NR	NR	Batch [100–400 mg/L, 50–500 ppb]	26
NH_2_‐MIL‐53(Fe)	EE2 (1.85 mg/g)	NR	Four cycles	NR	Batch [100–400 mg/L, 50–500 ppb]	26
Fe_3_O_4_@MlL‐101	E1 (80.6, 86.7 %) E2 (99.6, 84.3 %) E3 (82.0, 87.9 %)	NR	NR	River and lake water	Magnetic solid extraction [5 mg, 10 min, pH 7, 10 ppb]	27
Ce doped DUT‐52	E1 (286.9 mg/g, 92.1–96.1 %) E2 (291.6 mg/g, 82.1–91.9 %)	NR	Six cycles	Human urine	Dispersive solid phase extraction [10 mg, 3 min, 10–50 ng/mL]	28
Ca‐MIX	52.03 µg/g	pseudo‐second‐order (10 µg/L using 50 mg)	Five cycles (moderate)	NR	Batch adsorption [50 mg adsorbent, 2 h contact time, native pH]	29

In light of the adsorption experiments and overall assessment of capture efficiency and regenerability, MOF **3** stands out as the most promising material (Table 1). To gain structural insight into the host–guest interactions that may underlie the excellent SPE performance of MOF **3**, we determined the single‐crystal structure of MOF **3** loaded with 17β‐estradiol (E2), selected as a representative estrogen guest rather than as a universal model for all four hormones (**E2@3**). **E2@3** was obtained by soaking crystals of **3** in a saturated aqueous solution of E2 for 7 days (see Experimental Section). After this lapse of time, the crystal structure of **E2@3** could be determined by SCXRD measurements (vide infra, see Supporting Information details) –using synchrotron radiation at the I19 beamline of the Diamond Light Source (Table S6). (see Supporting Information details). Additionally, the chemical nature of **E2@3** was further established by C, H, N and TGA (Figure S6) analyses and PXRD measurements (Figure S7).

The crystal structure of **E2@3** is isomorphous with **3**, crystallizing in the *P*6_3_ chiral space group of the hexagonal system. Despite the persistent disorder of the **E2** molecules within the cage, plausible configurations and localizations were obtained via SCXRD (see Supporting Information for structural details). The guest molecules occupy the pores, where they are stabilized by supramolecular interactions mediated by the leucine‐derivative side chains (Figures 4, 5; S8 and S9). Refinement of the crystal structure reveals a 1:3 statistical occupancy (see Supporting Information for details and Figure S8). The packing of the **E2** guests appears to be governed by the limited polarity and significant steric bulk of the 17β‐estradiol molecule. Specifically, the molecules are held in place by weak C─H···C [C···C of 3.13(2)‐3.18(2) Å] and C─H···O water‐mediated supramolecular interactions [C···O of 2.70(2) Å] (Figures 5 and S9). The low loading of the guest is likely associated with its large molecular volume –characterized by the four‐ring steroid nucleus– and its hydrophobic nature. Nevertheless, the crystallographic model provides a representative snapshot of how E2 can be accommodated within the pores of MOF **3**. The steroid framework, comprising three six‐membered rings and one five‐membered ring, is oriented such that the 17β‐hydroxyl group points toward the center of the pores. This orientation places the hydroxyl group distal to the walls of the host matrix **3**, where it is likely stabilized by hydrogen bonding with unresolved lattice water molecules. The presence of the hydroxyl group at the C17 position in the β‐orientation (critical for its biological activity) was confirmed by the guest model. While statistical disorder precluded the direct localization of the C3‐hydroxyl group on **E2** (see Supporting Information), the orientation of the aromatic six‐membered ring suggests the formation of hydrogen bonds with the oxamate fragments of the leumox ligand (Figure S10). Overall, the **E2@3** crystal structure provides a representative structural picture of the host–guest interactions that can be established within MOF **3**. These observations are consistent with a combination of hydrophobic confinement, weak dispersive interactions, and adaptive pore flexibility contributing to estrogen capture under SPE conditions, although the relative importance of these interactions for the different estrogens has not been directly established.

**FIGURE 4:**
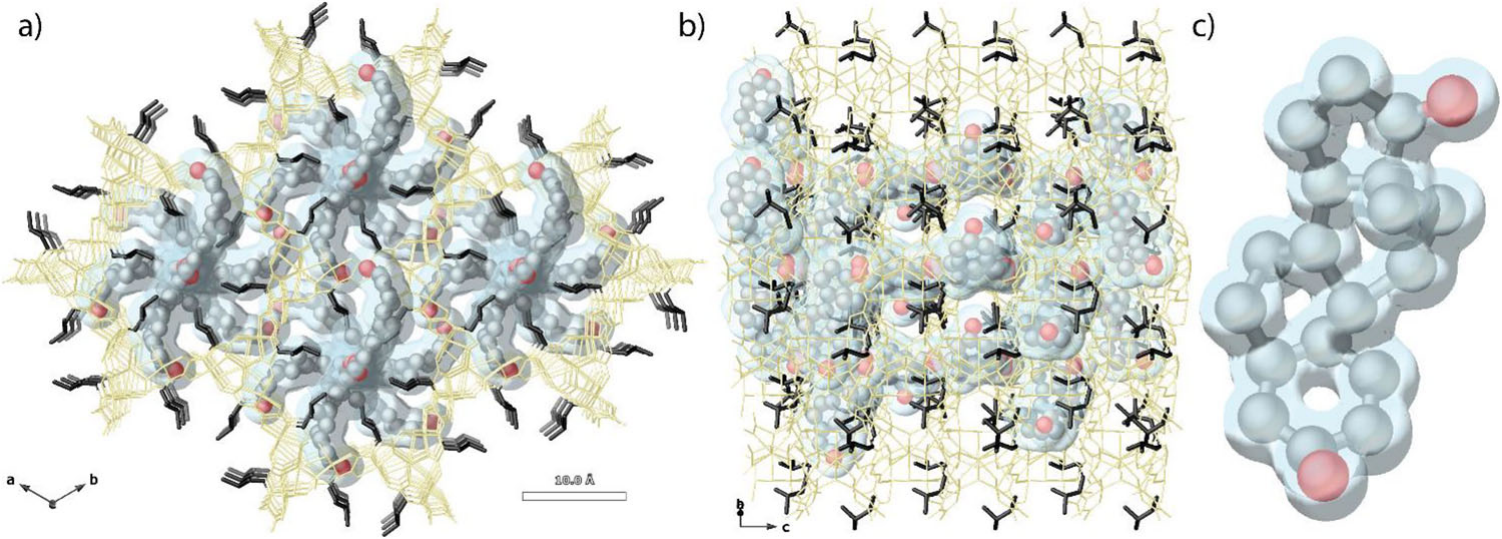
Views of the 3D open framework of **E2@3** along the *c* (a) axis and *b* (b) axis (crystallization water molecules omitted for clarity). The 3D network is represented as gold sticks, except for the *L*‐leucine residues, which are depicted as dark gray thick sticks. (c) Perspective view of the 17β‐estradiol (E2) guest molecule. Guest E2 molecules are shown as gray (carbon) and red (oxygen) spheres, with a blue surface added for enhanced visualization.

**FIGURE 5:**
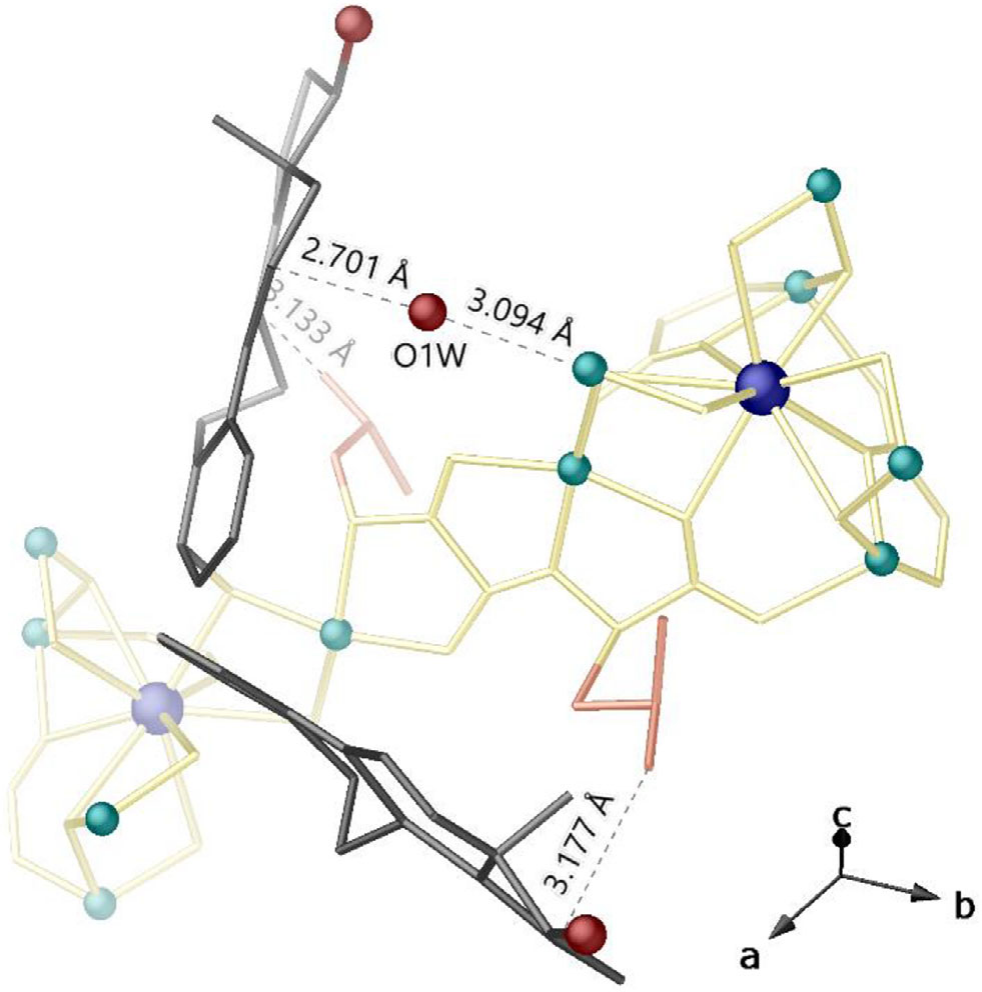
Details of host‐guest interactions in **E2@3**. Spot of weak CH_3_···CH involving leucine residue methyl groups (CH_3_···CH and CH···O_Water_ are represented by dashed lines). Copper, calcium, and oxygen of the E2 are represented by cyan, blue, and red spheres, respectively, whereas the ligands and guest molecules are depicted as gold and gray sticks, respectively.

The crystallographic analysis therefore provides direct structural evidence that steroidal estrogens can be accommodated within the flexible hydrophobic pores of MOF **3** through a combination of hydrogen bonding, dispersive interactions, and geometric confinement. These observations are consistent with the excellent adsorption and regeneration performance observed under SPE conditions.

## 3 Conclusion

In summary, we have demonstrated that amino‐acid–derived oxamidato‐based MOFs constitute a highly effective platform for the rapid, simultaneous, and selective capture of multiple estrogenic hormones from aqueous media. Among the evaluated materials, a leucine‐functionalized MOF (**3**) exhibits exceptional performance, combining fast uptake kinetics, near‐quantitative removal of estrone, 17β‐estradiol, estriol, and 17α‐ethinylestradiol in a single loading step, and outstanding regeneration. The material maintains its capture efficiency over multiple reuse cycles and performs equally well in real river‐water samples, underscoring its robustness under environmentally relevant conditions. The superior behavior of this MOF is attributed to the unique combination of a resilient inorganic backbone and flexible, confined amino‐acid residues that adapt to guest molecules, enabling efficient host–guest recognition while avoiding irreversible binding. Importantly, the crystal structure of a host–guest adsorbate (**E2@3**) provides direct visualization of estrogen encapsulation within the framework pores, offering structural insight into the interactions (weak but efficient) governing the observed capture and release properties.

Overall, this work demonstrates that flexible, amino acid‐derived MOFs can simultaneously achieve ultrafast kinetics, high efficiency, and exceptional regenerability, addressing key limitations of current adsorbents and opening new opportunities for analytical solid‐phase extraction and cartridge‐based water purification technologies. Although the present study was carried out under SPE conditions, the excellent stability, rapid adsorption kinetics, and regenerability of these materials also highlight their potential for future continuous‐flow separation systems. Further studies addressing large‐scale synthesis, techno‐economic aspects, and process engineering will be necessary to assess their implementation in full‐scale water treatment applications.

## Conflicts of Interest

The authors declare no conflicts of interest.

## Supporting information




**Supporting File**: chem71554‐sup‐0001‐SuppMat.pdf

## Data Availability

The data that support the findings of this study are available from the corresponding author upon reasonable request.
