# Supplementary material for: Rapid and Reusable Amino Acid–Derived MOFs for the Removal of Estrogenic Hormones From Real Water Matrices
Source: Chemistry. 2026 Aug 13;32(41):e71554. doi: 10.1002/chem.71554 (PMC13730000; doi:10.1002/chem.71554)
Supplement: Supplementary file 1 [file CHEM-32-e71554-s001.pdf]

Supporting Information (SI) for the manuscript:

Rapid and Reusable Amino Acid–Derived MOFs for the Removal of Estrogenic Hormones from Real Water Matrices

Lidia García,[a] Alejandro Cortés-Villena,[b] Héctor Martínez Pérez-Cejuela,*,[b] José Manuel Herrero-Martínez,[b] Jesús Ferrando-Soria,[a] Thais Grancha,[a] Donatella Armentano,*,[c] and Emilio Pardo*,[a]

[a] Instituto de Ciencia Molecular (ICMol), Universidad de Valencia, 46980 Paterna, Valencia, Spain.

[b] Department of Analytical Chemistry, University of Valencia, c/Dr. Moliner, 50, 46100-Burjassot, Valencia, Spain.

[c] Dipartimento di Chimica e Tecnologie Chimiche (CTC), Università della Calabria, Rende 87036, Cosenza, Italy.

Experimental Section

Materials. All chemicals were of reagent grade quality. They were purchased from commercial sources and used as received.

Preparation of MOFs and MTV-MOFs 1-6: $Ca^{II}Cu^{II}_6[(S,S)\text{-alamox}]_3(OH)_2(H_2O)\} \cdot 32H_2O$[1] (**1**), $\{Ca^{II}Cu^{II}_6[(S,S)\text{-methox}]_3(OH)_2(H_2O)\} \cdot 16H_2O$[2,3] (**2**) and $\{Ca^{II}Cu^{II}_6[(S,S)\text{-leumox}]_3(OH)_2(H_2O)\} \cdot 11H_2O$[4] (**3**) and $\{Sr^{II}Cu^{II}_6[(S,S)\text{-threonine}]_3(OH)_2(H_2O)\} \cdot 36H_2O$[5] (**4**), as well as the two multivariate (MTV) MOFs – incorporating two different amino acid–derived ligands within the same structure– with formulas $\{Ca^{II}Cu^{II}_6[(S,S)\text{-methox}]_{1.5}[(S,S)\text{-serimox}]_{1.50}(OH)_2(H_2O)\} \cdot 30H_2O$[6] (**5**) and $\{Sr^{II}Cu^{II}_6[(S,S)\text{-methox}]_{1.5}[(S,S)\text{-Mecysmox}]_{1.50}(OH)_2(H_2O)\} \cdot 36H_2O$[7,8] (**6**) [where alamox = bis[(S)-alanine]oxalyl diamide; serimox = bis[(S)-serine]oxalyl diamide; methox = bis[(S)-methionine]oxalyl diamide; threomox = bis[(S)-threonine]oxalyl diamide; leumox = bis[(S)-leucine]oxalyl diamide; and Mecysmox = bis[(S)-methylcysteine]oxalyl diamide] have been prepared, as polycrystalline powders, as previously reported. On the other side, aiming at crystallographic determination of host-guest adsorbates, well-shaped hexagonal prisms of **3**, suitable for SCXRD, were obtained by slow diffusion methods as reported previously.[4]

Preparation of (17β-estradiol)$_{0.5}$@$\{Ca^{II}Cu^{II}_6[(S,S)\text{-leumox}]_3(OH)_2(H_2O)\} \cdot 6H_2O$ (E2@3): Well-shaped hexagonal prisms of **E2@3**, suitable for SCXRD, could be obtained by soaking crystals of MOF **3** (*ca.* 5.0 mg) for a week in saturated aqueous solutions containing E2 (recharging fresh saturated solutions daily). After this period, they were isolated by filtration, air-dried and characterized by SCXRD, PXRD, C, H, N, and TGA analyses to obtain their chemical formula. Anal.: calcd. for **E2@3**: $C_{33}Cu_6CaH_{64}N_6O_{26}$ (1382.2): C, 28.68; H, 4.67; N, 6.08% Found: C, 28.62; H, 4.57; N, 6.11%; IR (KBr): ν = 1603 and 1611 cm^{-1} (C=O).

Maximum uptake capacity of 3: The maximum uptake capacity of MOF **3**, for each estrogen, was determined. For that, *ca.* 100 mg of **3** were soaked in water/methanol (5:1, v/v) saturated solutions of the corresponding estrogen [estrone (E1), 17β-estradiol (E2), estriol (E3), and 17α-ethinylestradiol (EE2)] during five days. Each saturated solution was replaced every 12 hours in order to ensure the maximum loading. After this period, each hybrid material was filtered off and washed with water. The estrogen contents of each material was determined by the combination of both C, H, N, S analyses (*vide infra*) and gravimetry.

C, H, N analyses: **3 + E1:** Anal. for $C_{72.24}Cu_6CaH_{100.96}N_6O_{24.36}$ (1864.6): C, 46.53; H, 5.46; N, 4.51%. **3 + E2:** Anal. for $C_{81.96}Cu_6CaH_{117,28}N_6O_{25.44}$ (2015.03): C, 48.85; H, 5.87; N, 4.17%. **3 + E3:** Anal. for $C_{68.28}Cu_6CaH_{99}N_6O_{25.38}$ (1831.38): C, 44.78; H, 5.45; N, 4.59%. **3 + EE2:** Anal. for $C_{77,8}Cu_6CaH_{106,96}N_6O_{24,58}$ (1940.9): C, 48.14; H, 5.55; N, 4.33%.

Physical Techniques: Elemental (C, H, S, N) analyses were performed at the Microanalytical Service of the Universitat de València. FT–IR spectra were recorded on a Perkin-Elmer 882 spectrophotometer as KBr pellets. The thermogravimetric analyses were performed on crystalline samples under a dry N_2 atmosphere with a Mettler Toledo TGA/STDA 851e thermobalance operating at a heating rate of 10 ºC min^{-1}.

The N_2 adsorption-desorption isotherms at 77 K were carried out on crystalline samples of **1-6** with a Belsorp MINI X instrument. Samples were activated at 100 °C under reduced pressure (10^{-6} Torr) for 16 h prior to carrying out the sorption measurements.

Analytical experiments:

- Instrumentation:

For the solid-phase extraction (SPE) protocols, a manifold (VacElut) with twelve positions (Agilent Technologies, Waldbronn, Germany) and a pump for vacuum N938 Laboport (KNF, Freiburg, Germany) were used. For HPLC conditions, an 1100 series liquid chromatograph (Agilent Technologies) provided with a quaternary pump, a degasser, a thermostated column compartment, an automatic sampler, and equipped with diode-array (DAD) and fluorescence (FLD) detection. Separation was accomplished with a Kinetex EVO C18 (150 × 4.6 mm, 5 µm particle size, Phenomenex). The mobile phases consisted of water (A) and MeCN (B) at 1 mL min^{-1}. Gradient mode was used to achieve the chromatographic separation as follows: Initial gradient of 25% B was set, then at 3 min B was set at 50%, which was kept constant up to 7 min. After that, B was increased to 95% at min 7.5 and kept for 2 more min, when the gradient was set to the initial conditions. The injection volume was 25 µL, no temperature control was needed, and the detection DAD wavelength was 280 nm while FLD wavelengths were fixed at 285 and 310 nm for excitations and emission, respectively.

- Capture experiments:

To conduct capture studies, an SPE protocol was implemented, which can be summarized as follows. SPE cartridges were prepared as previously described. Previous to sample loading, the cartridge was conditioned with 2 mL of methanol (CH_3OH) and 2 mL of water. Then, aqueous standard mixtures (1 mL) containing target drugs were loaded onto the SPE cartridge, followed by a 2 mL water washing. Elution was performed with 1 mL of CH_3OH in 15 seconds, and the cartridge was reconditioned with 2 mL each of CH_3OH and water. All SPE fractions were filtered through a 0.22 µm polytetrafluoroethylene (PTFE) membrane and injected into the HPLC-DAD system. The integrity of the MOFs after each capture experiment was repeatedly verified

through ICP-MS analysis, which confirmed that no metal leaching occurred in any of the experiments.

- Reusability experiments:

To perform reusability studies, the same SPE protocol as for capture experiments was implemented (see previous section), but the elution fractions from protocols nº 1-3-5-10 were analyzed after each cycle. The stability of the MOF after each experiment was confirmed by means of its PXRD pattern. Powdered activated carbon (PAC) used for comparison was purchased from Sigma-Aldrich (CAS: 7440-44-0) and used as received.

- Reagents and materials:

Standard solutions of estrone, estriol, 17β-estradiol and 17β-ethinylestradiol were obtained from Sigma (Stenheim, Germany). See details and structures in Scheme S1. All the solvents (*e.g.*, methanol (CH_3OH) or MeCN and others) were all HPLC grade and purchased from VWR International Eurolab (Barcelona, Spain). Nanopure water was purified in a Crystal B30 EDI Adrona deionizer (Riga, Latvia). Other non-specific reagents were of analytical grade unless otherwise stated. SPE propylene cartridges of 1mL (internal volume) and their respective frits (1/16′, 20 μm) were provided by Análisis Vínicos (Tomelloso, Spain). A multicomponent stock solution was prepared at a 10 mg L^{-1} concentration level in CH_3OH, and stored at -20 °C in an amber glass vial. Standard mixtures were prepared by dilution of this stock solution. Calibration curves ranging from 0.5 to 50 µg L^{-1} were also prepared by diluting this multicomponent stock solution in water.

X-ray crystallographic data collection and structure refinement: A crystal of **E2@3** with dimensions 0.06 x 0.05 x 0.05 was selected and mounted on a MITIGEN

holder in Paratone oil and very quickly placed on a liquid nitrogen stream cooled at 100 K, to avoid the possible degradation upon dehydration or exposure to air. Diffraction data were collected using synchrotron radiation at I19 beamline of the Diamond Light Source at λ = 0.6889 Å. The data were processed through xia2 software.[9–13] The structures were solved with the SHELXS structure solution program, using the Patterson method. The model was refined with version 2019/1 of SHELXL against F^2 on all data by full-matrix least squares.[14–16]

As reported in the main text, the robustness of the 3D network, allowed the resolution of the crystal structure of host-guest adsorbate **E2@3**, being their crystals suitable for X–ray diffraction, even over one–step process, after a crystal–to–crystal transformation. For these reasons it is reasonable to observe a diffraction pattern sometimes affected by expected internal imperfections of the crystals [likely at the origin of some Alert level A in checkcifs related to U(eq) value of some atoms] and thus a quite expected difficulty to perform a perfect correction of anisotropy, mainly affected by highly flexible thioether chains as terminal moiety (vide infra).

All non-hydrogen atoms of the networks were refined anisotropically, except the leucine-derived chains from the Leumox ligand and all highly dynamically disordered atoms of guest molecules. The latter have been refined with a lot of restraints and constraints of rigid groups. For such kind of single crystals, the lower data quality for adsorbates, embedding highly disordered guest molecules, makes the use of constraints and especially restraints essential. The use of some bond lengths restraints applied on atoms belonging to highly dynamic moieties, has been reasonably imposed and related to the expected thermal motion, likely depending on the large pore size of the frameworks (FLAT, DFIX, DANG, SIMU, DELU and ISOR). Aminoacidic chains have been systematically refined with restrains on C-C bond lengths. In their

refinements some further restraints, to make the refinement more efficient, have been applied. For instance, SAME components have been restrained to be like other related atoms, using SIMU for disordered sections or fixed ADP (after 10 cycles of refinements) for group of atoms of the guest molecules expected to have essentially similar ADPs.

Due to the impressive statistical disorder, other than the dynamical one, hydrogen atoms were not defined either for the network nor for the guest molecules. In fact, it is often expected that guest molecules are severely disordered, as a direct consequence of their high thermal motion and exhibited statistical disorder. It should be also underlined that our best model has taken into account the most persistence conformations –within the additional complication– that even a low percentage, not taken into account, such as a 10% of a whole Estradiol guest molecule, is still a significant amount of electron density, and whole-molecule disorder lurks everywhere in the inky shadows of structure refinement, affecting quality of the model.

In the **E2@3** crystal structure refinement, the occupancies of the guests in the pores were fixed at 0.166, in good agreement with bulk CHN analysis. Unfortunately, thermal motion has also not been refined for disordered sites, for which after a first four cycles of refinements it was blocked. We strongly believe that it is the more reliable way to attempt to define loading instead of considering merely thermal factors, which can be affected by a lot of issues above all severe disorders.

As stated above and as expected, estradiol molecules are statistically and thermally disordered. Their fragments are severely disordered exhibiting three sets of possible orientations of the guest molecules as detailed in Figure S8. This imposed the use of further constraints to block thermal parameters of atoms of those fragments representing terminal groups during the final refinement. The partial overlap between different

orientations at atom sites, together with thermal disorder of the leucine-derived chains of ligand makes the final refinement really challenging (Figure S10) without reaching a suitable level of convergence. It is a poorly behaved structure, here parameters related to disordered fragments remain constantly oscillating even if appropriate restraints have been introduced to stabilise oscillating parameters.

A summary of the crystallographic data and structure refinement for the **E2@3** crystal structure is given in Table S5. The comments for the alerts A and B are reported in the CIFs using the validation response form (vrf). CCDC reference number is 2541322.

The final geometrical calculations on free voids and the graphical manipulations were carried out with PLATON[17,18] implemented in WinGX,[19] and CRYSTAL MAKER[20] programs, respectively.

X-ray Powder Diffraction Measurements: Fresh polycrystalline samples of **1-6**, MOF **3** after 10 resuses, and also the host-guest adsorbate **EE2@3**, were introduced into 0.5 mm borosilicate capillaries prior to being mounted and aligned on an Empyrean PANalytical powder diffractometer, using Cu Kα radiation (λ = 1.54056 Å). For each sample, five repeated measurements were collected at room temperature (2θ = 2–60°) and merged in a single diffractogram.

Table S1. Removal efficiencies (%) of MOFs **1-6** from an aqueous solution (pH 7.0) containing a mixture of selected hormones E1, E2, E3 and EE2 (2 mg L^{-1} for each hormone). The values shown in parentheses indicate the relative standard deviation (%RSD).

**Estrogen**	**% Removal efficiency (%RSD)**					
	**MOF 1**	**MOF 2**	**MOF 3**	**MOF 4**	**MOF 5**	**MOF 6**
E1[a]	48 (4)	71 (2)	92 (2)	53 (2)	59 (3)	96 (1)
E2[b]	37 (2)	89 (3)	93 (1)	77 (1)	82 (2)	98 (2)
E3[c]	22 (2)	91 (2)	96 (3)	85 (2)	91 (1)	99 (2)
EE2[d]	53 (2)	89 (3)	94 (2)	72 (3)	89 (3)	98 (2)

[a]E1 = estrone, [b]E2 = 17β-estradiol, [c]E3 = estriol, [d]EE2 = 17α-ethinylestradiol.

Table S2. Elution (%) of E1, E2, E3 and EE2 contained in loaded MOFs **1-6** after percolating 1 mL of CH_3OH through the cartridge. The values shown in parentheses indicate the relative standard deviation (%RSD).

**Estrogen**	**% Recovery (%RSD)**					
	**MOF 1**	**MOF 2**	**MOF 3**	**MOF 4**	**MOF 5**	**MOF 6**
E1[a]	49 (1)	39 (4)	90 (1)	29 (3)	26 (1)	34 (2)
E2[b]	46 (3)	47 (1)	91 (2)	45 (2)	35 (2)	51 (3)
E3[c]	43 (1)	43 (3)	93 (1)	40 (1)	37 (3)	51 (4)
EE2[d]	67 (3)	47 (2)	88 (2)	42 (2)	34 (4)	52 (1)

[a]E1 = estrone, [b]E2 = 17β-estradiol, [c]E3 = estriol, [d]EE2 = 17α-ethinylestradiol.

Table S3. Removal efficiencies (%) of MOF **3** for E1, E2, E3, and EE2 over 10 consecutive capture cycles from an aqueous solution (pH 7.0) containing a mixture of E1, E2, E3, and EE2 (2 mg L^{-1} of each hormone). Values in parentheses represent the relative standard deviation (%RSD).

**Estrogen**	**% Removal efficiency (%RSD)**			
	**1st cycle**	**3rd cycle**	**5th cycle**	**10th cycle**
E1[a]	98 (1)	96 (3)	98 (1)	98 (2)
E2[b]	97 (2)	96 (2)	98 (2)	97 (3)
E3[c]	99 (3)	98 (2)	99 (2)	99 (2)
EE2[d]	96 (1)	96 (1)	97 (1)	97 (4)

[a]E1 = estrone, [b]E2 = 17β-estradiol, [c]E3 = estriol, [d]EE2 = 17α-ethinylestradiol.

Table S4. Removal Capture efficiency (%) of MOF **3** for E1 (red), E2 (blue), E3 (green) and EE2 (orange) from an aqueous solution from the Turia river spiked with 2 mg L^{-1} of each estrogen. The values shown in parentheses indicate the relative standard deviation (%RSD).

**Estrogen**	**% Recovery (%RSD)**
E1[a]	98 (1)
E2[b]	99 (1)
E3[c]	99 (1)
EE2[d]	99 (2)

[a]E1 = estrone, [b]E2 = 17β-estradiol, [c]E3 = estriol, [d]EE2 = 17α-ethinylestradiol.

Table S5. Maximum loading capacities of MOF **3** for estrogens E1, E2, E3 and EE2 determined by gravimetric measurements and CHN elemental analyses.

**Estrogen**	**Mass of 3 (g)**	**Mass of 3 + estrogen (g)**	**C (%)**	**H (%)**	**N (%)**	**Estimated estrogen molecules[a] per formula unit**
E1	0.097	0.1293	46.53	5.46	4.51	1.68
E2	0.103	0.1459	48.85	5.87	4.17	2.22
E3	0.100	0.1299	44.78	5.45	4.59	1.46
EE2	0.099	0.1367	48.14	5.55	4.33	1.79

[a]Estimated estrogen molecules per $Ca^{II}Cu^{II}_6$ unit.

Table S6. Summary of Crystallographic Data for **E2@3**.

Compound	**E2@3**
Formula	$C_{42}Cu_6CaH_{76}N_6O_{27}$
M (g mol^{-1})	1518.40
λ (Å)	0.6889
Crystal system	hexagonal
Space group	$P6_3$
a (Å)	17.66800(14)
c (Å)	13.1005(2)
V (Å^3)	3541.55(8)
Z	2
ρ_{calc} (g cm^{-3})	1.424
μ (mm^{-1})	1.698
T (K)	100
θ range for data collection (°)	1.290 to 25.993
Completeness to θ = 25.0	100%
Measured reflections	48791
Unique reflections (Rint)	5102 (0.0483)
Observed reflections [$I > 2\sigma(I)$]	3906
Goof	2.096
Absolute structure parameter (Flack)	0.47(3)
R^a [$I > 2\sigma(I)$] (all data)	0.1732 (0.1894)
wR^b [$I > 2\sigma(I)$] (all data)	0.4419 (0.4668)
CCDC	2541322

^{a} $R = \sum(|F_o| - |F_c|)/\sum|F_o|$. ^{b} $wR = [\sum w(|F_o| - |F_c|)^2/\sum w|F_o|^2]^{1/2}$.

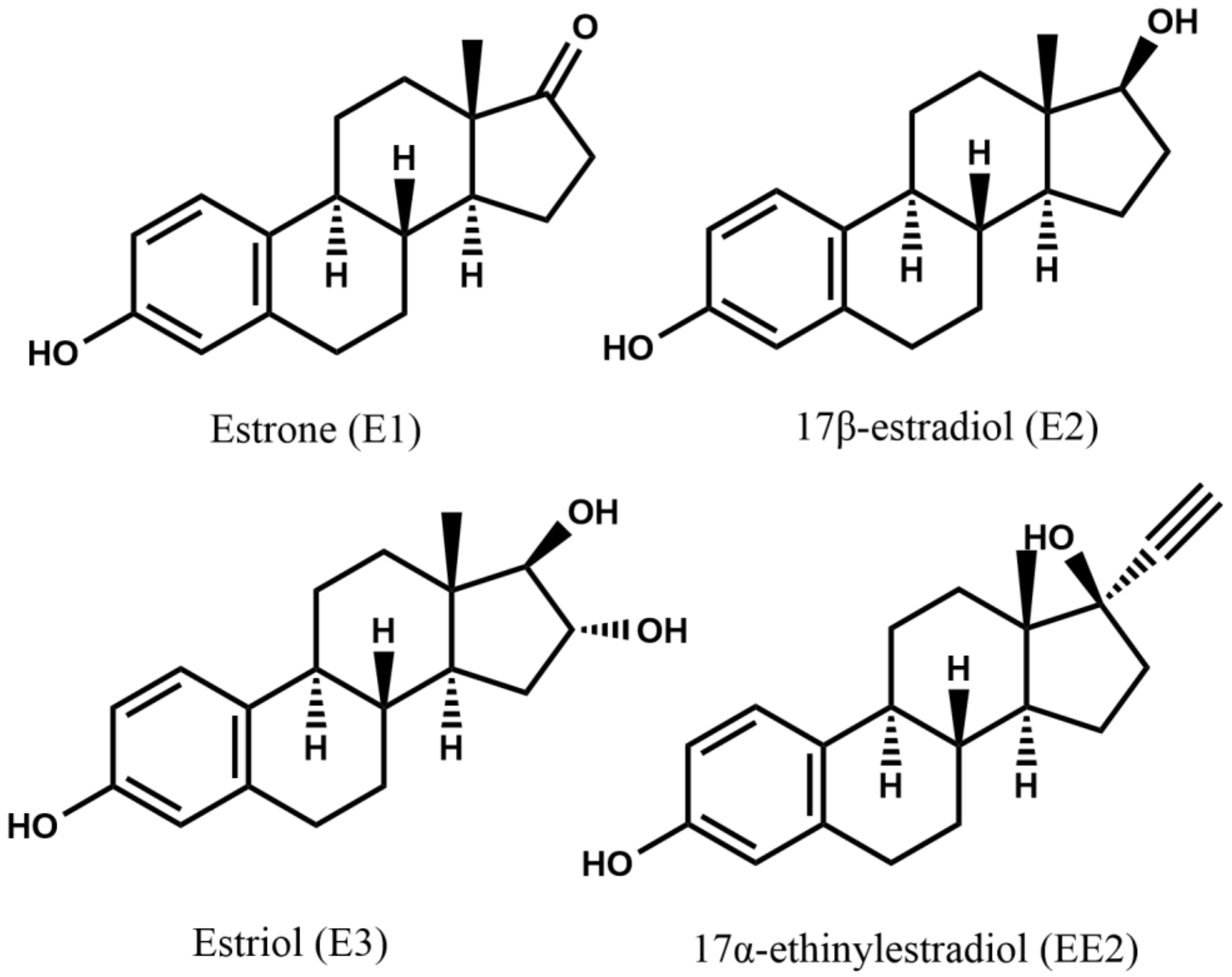

Scheme S1. Chemical structures of estrone (E1), 17β-estradiol (E2), estriol (E3), and 17α-ethinylestradiol (EE2).

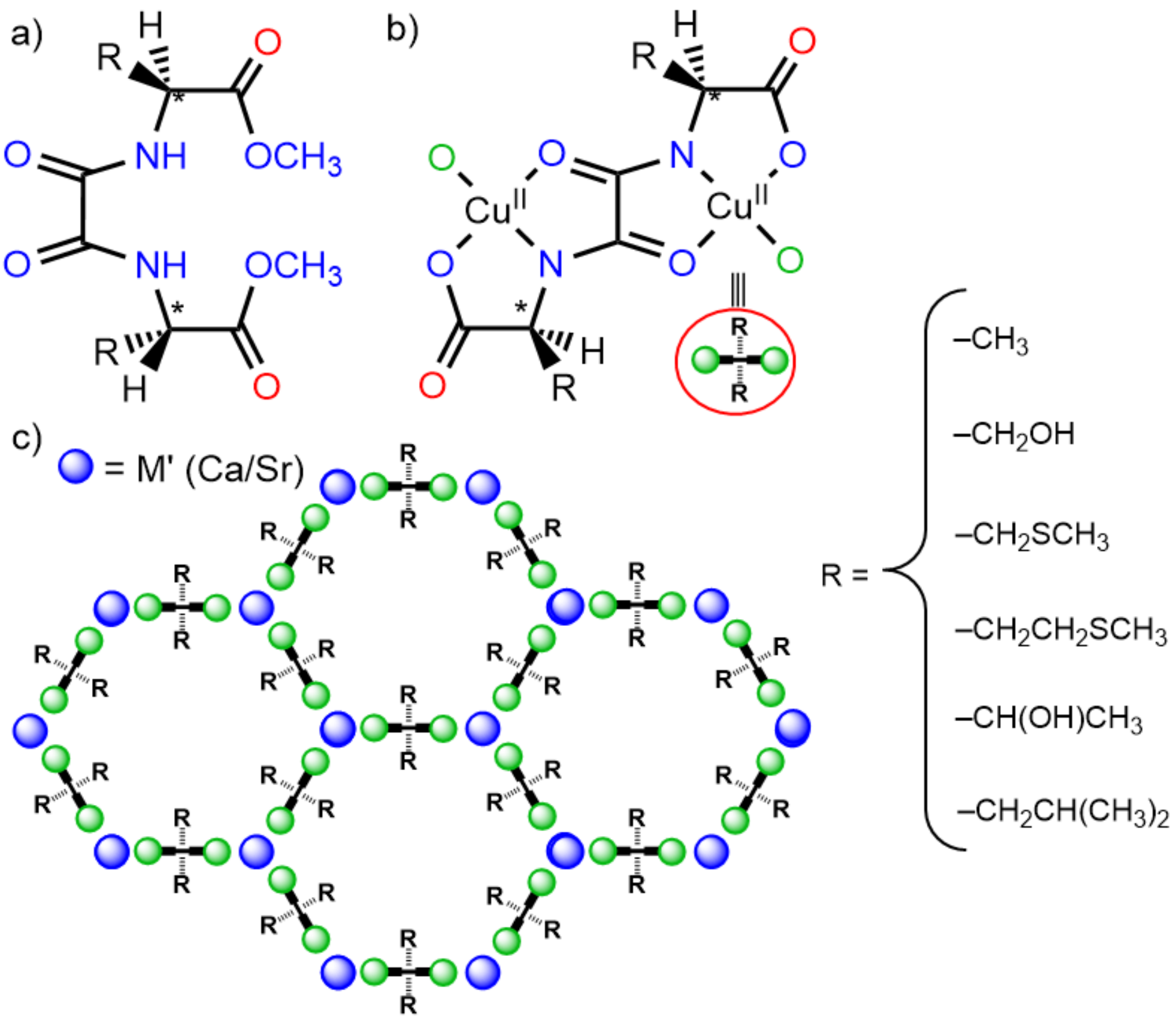

Scheme S2. Chemical structures of the amino acid-based oxamidato ligands (a) and dicopper(II) precursor complexes (b), as well as a schematic representation of the MOF hexagonal network emphasizing the position of the amino acid residues (R = $–CH_3$, MOF **1**; R = $–CH_2CH_2SCH_3$, MOF **2**; R = $–CH_2CH(CH_3)_2$, MOF **3**; R = $–CH(OH)CH_3$, MOF **4**; R = $–CH_2CH_2SCH_3$ and $–CH_2OH$, MTV-MOF **5**; R = $–CH_2CH_2SCH_3$ and – CH_2SCH_3, MOF **6**).

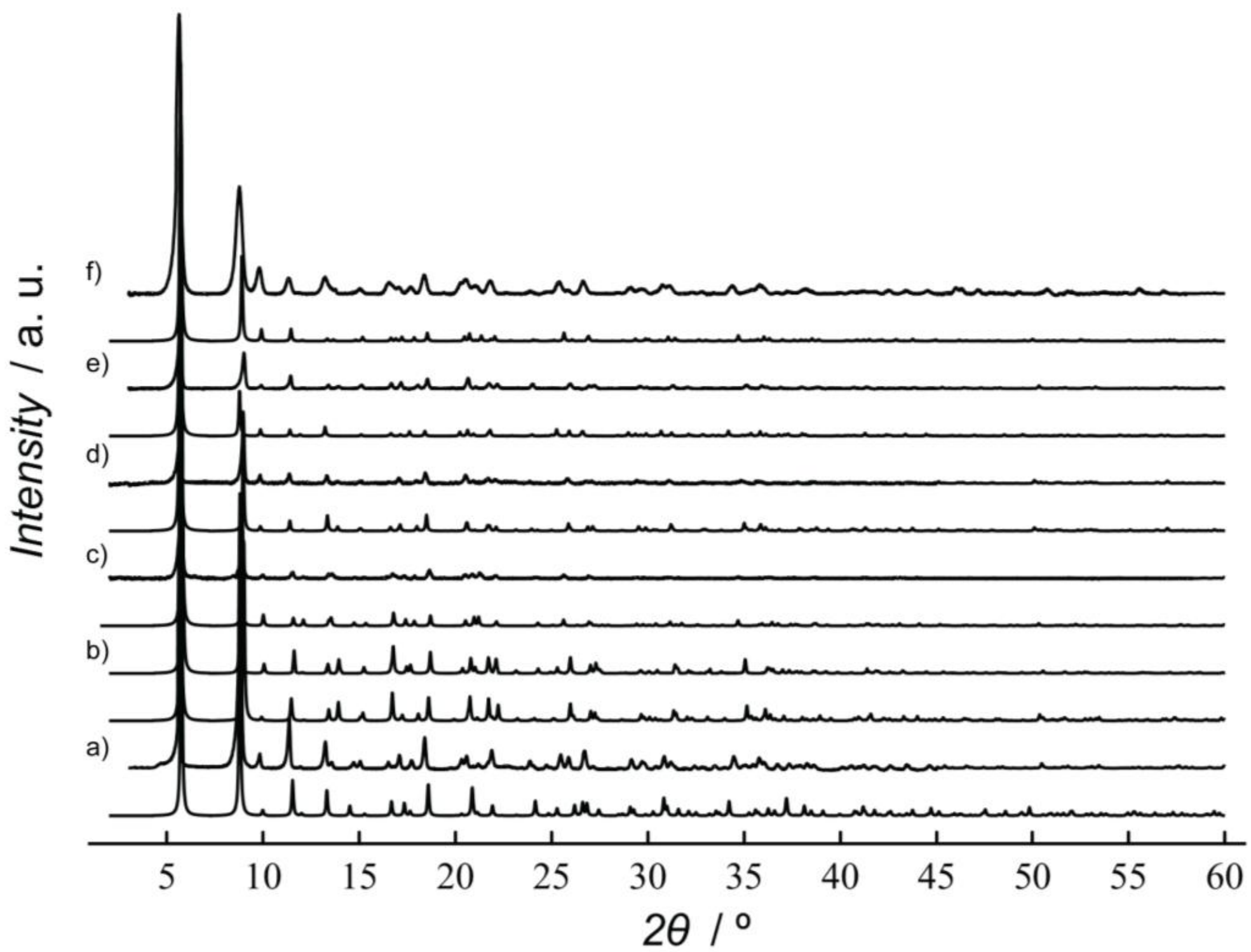

Figure S1. Theoretical (bottom) and experimental (top) PXRD patterns of MOFs **1** (a), **2** (b), **3** (c), **4** (d), **5** (e) and **6** (f) in the 2.0–60.0° range.

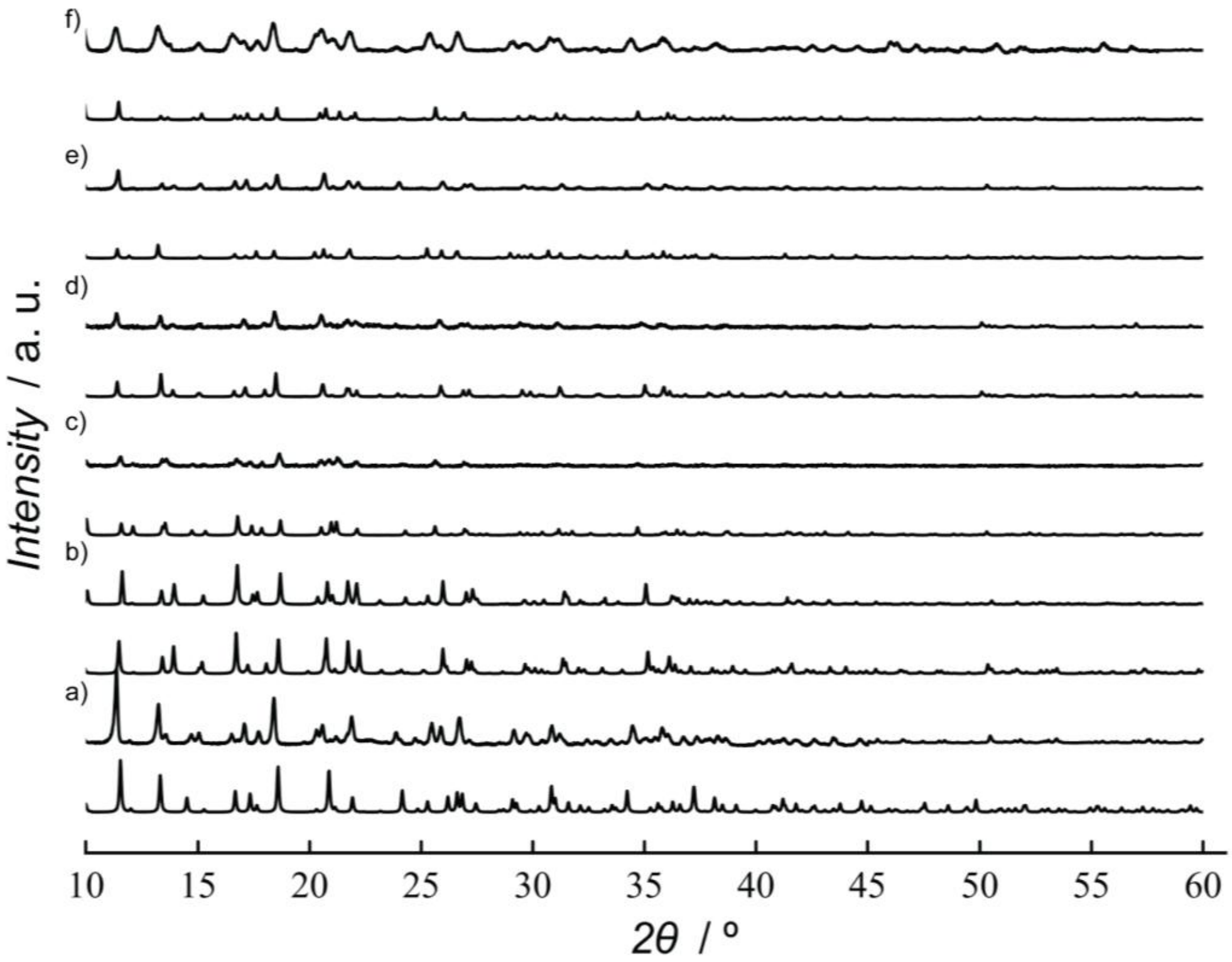

Figure S2. Theoretical (bottom) and experimental (top) PXRD patterns of MOFs **1** (a), **2** (b), **3** (c), **4** (d), **5** (e) and **6** (f) in the 10.0–60.0° range.

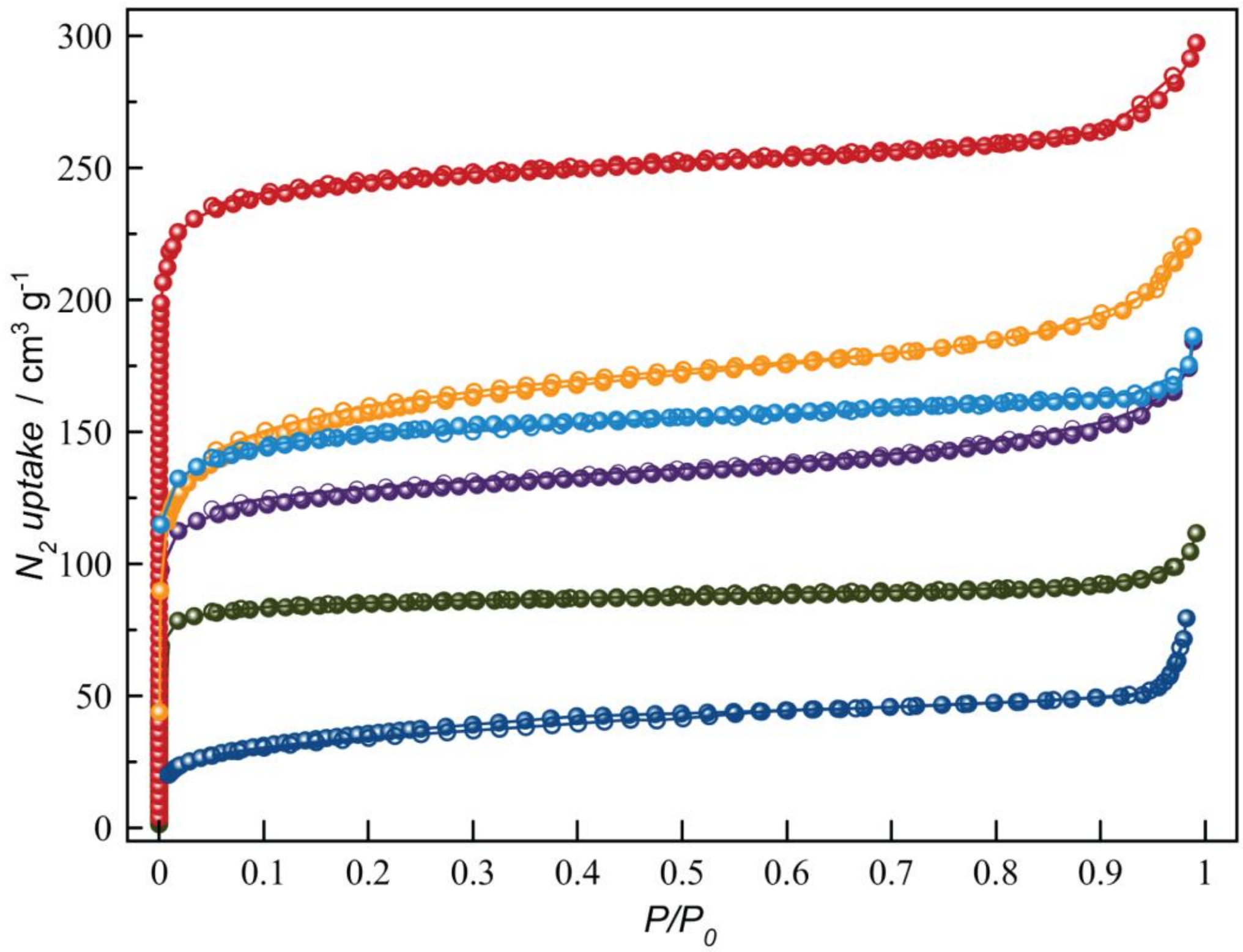

Figure S3. N_2 (77 K) adsorption isotherms for the activated compounds **1** (red), **2** (deep blue), **3** (green), **4** (orange), **5** (light blue) and **6** (magenta). Filled and empty symbols indicate the adsorption and desorption isotherms, respectively. The samples were activated at 100 °C under reduced pressure for 16 h prior to carry out the sorption measurements.

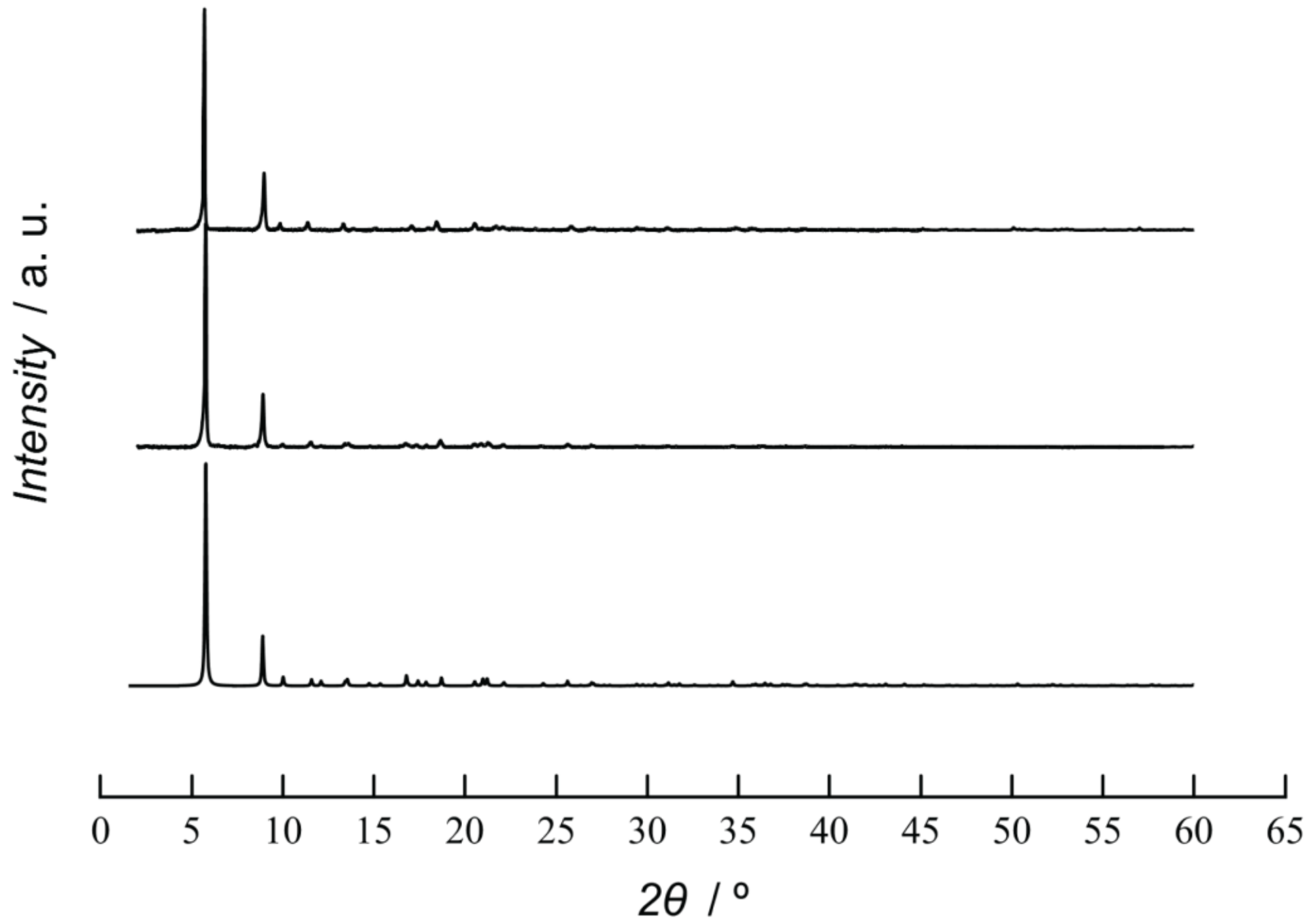

Figure S4. Theoretical (a) and experimental PXRD pattern of MOFs **3**, before (b) and after (c) 10 capture reuses, in the 10.0–60.0° range.

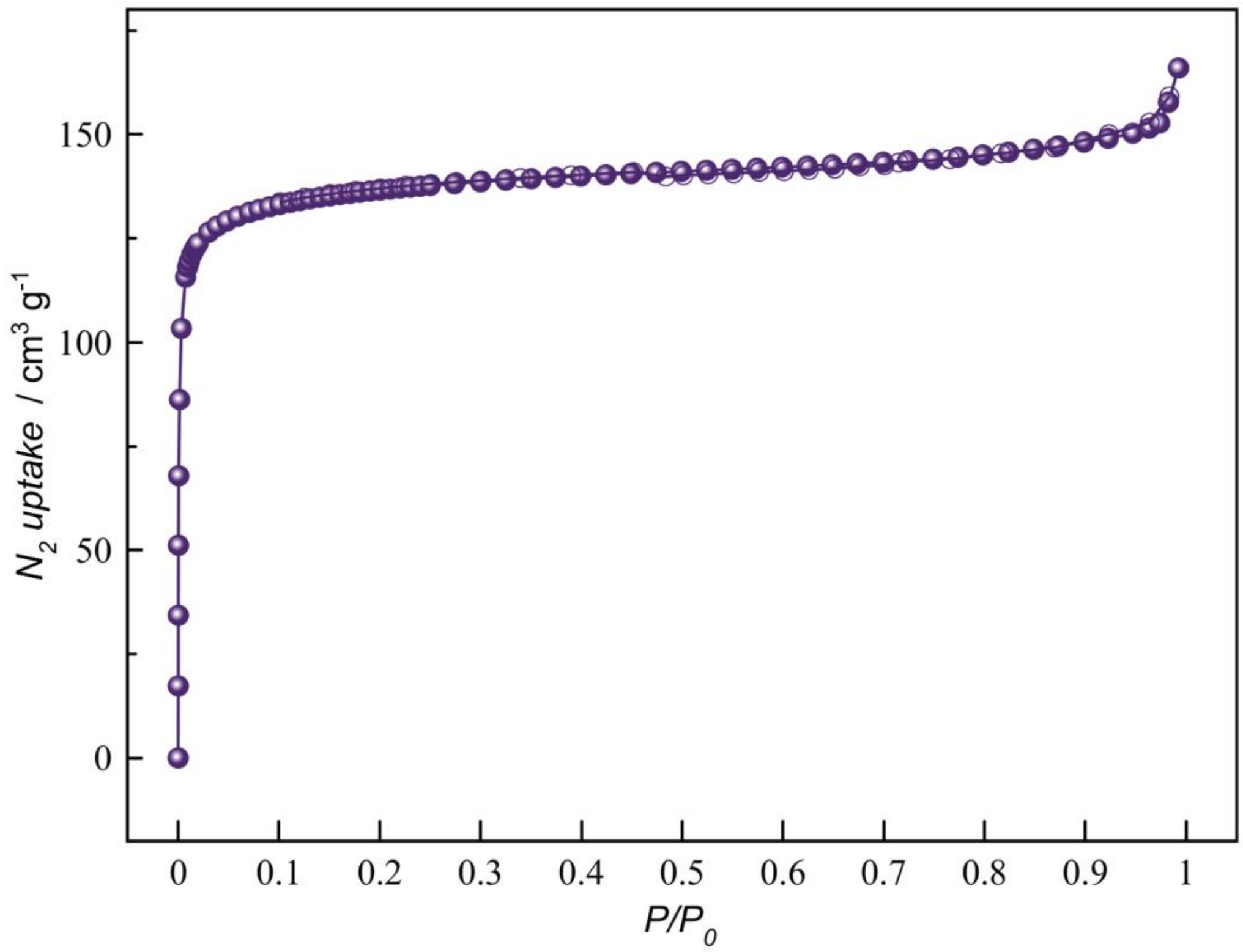

Figure S5. N_2 (77 K) adsorption isotherms for the activated MOF **3** after 10 sorption-desorption cycles. Filled and empty symbols indicate the adsorption and desorption isotherms, respectively. The samples were activated at 100 °C under reduced pressure for 16 h prior to carry out the sorption measurements.

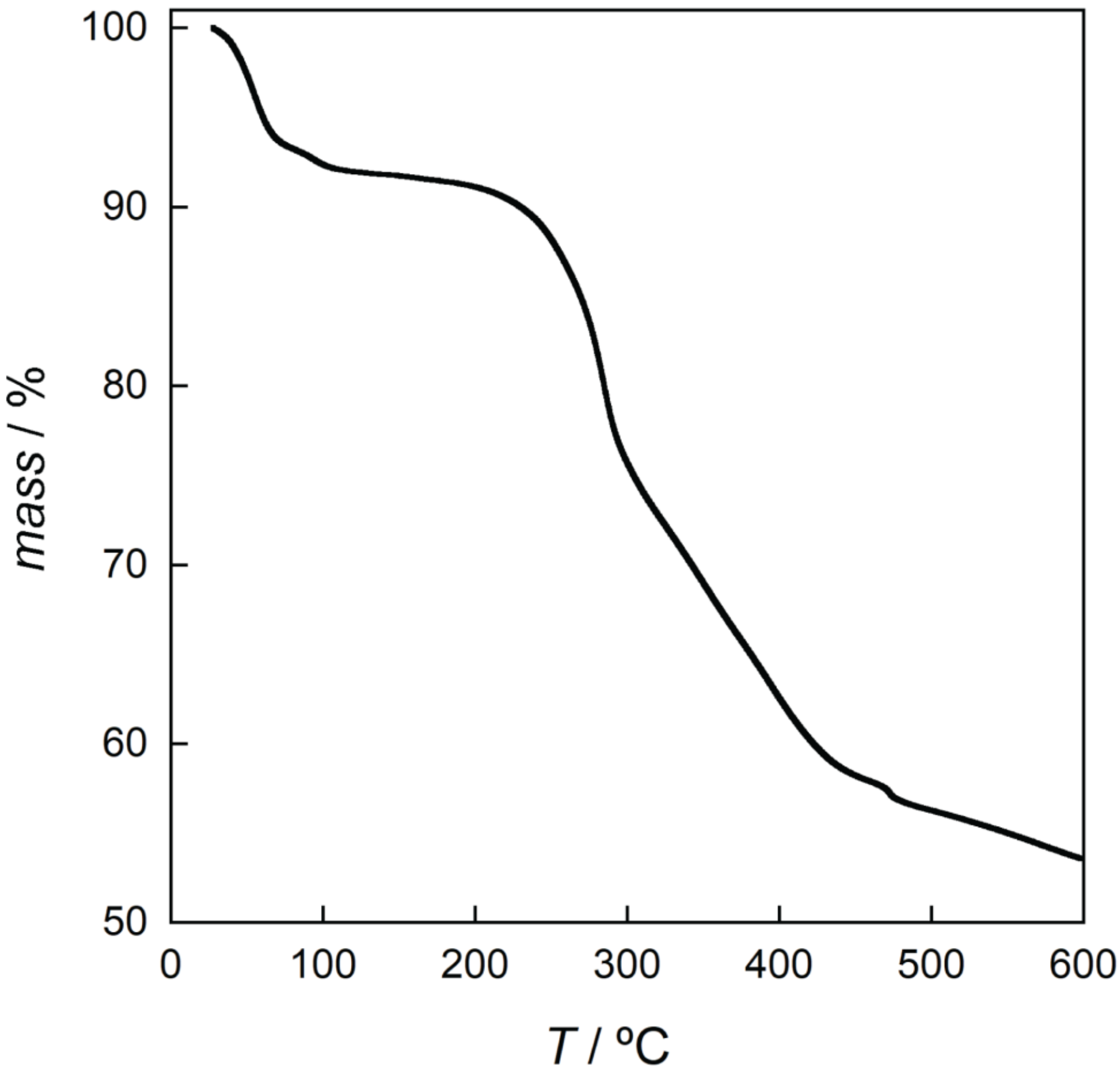

Figure S6. Thermo-Gravimetric Analysis (TGA) of **E2@3** under dry N_2 atmosphere.

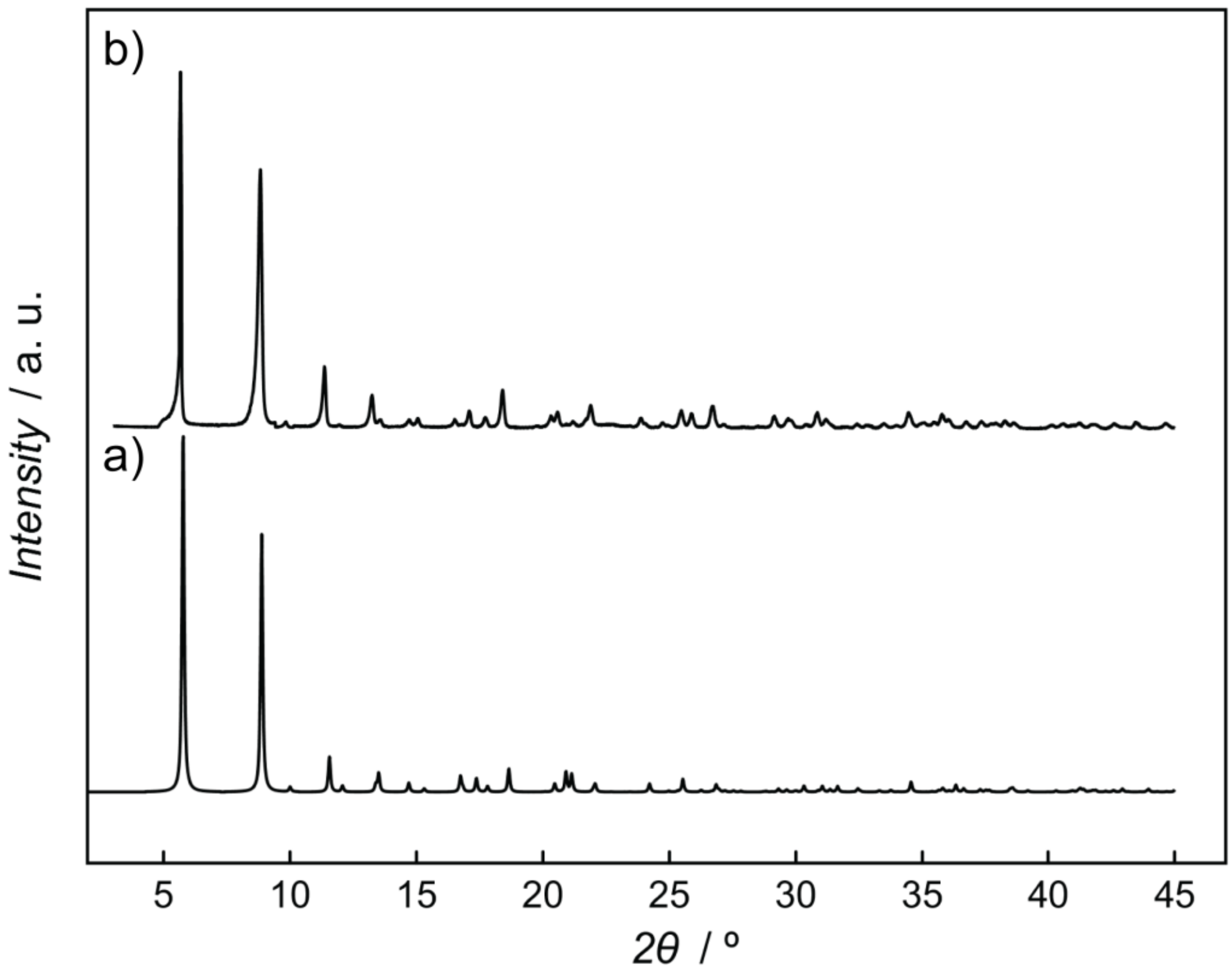

Figure S7. Theoretical (a) and experimental PXRD pattern of **E2@3** in the 3.0–45.0° range.

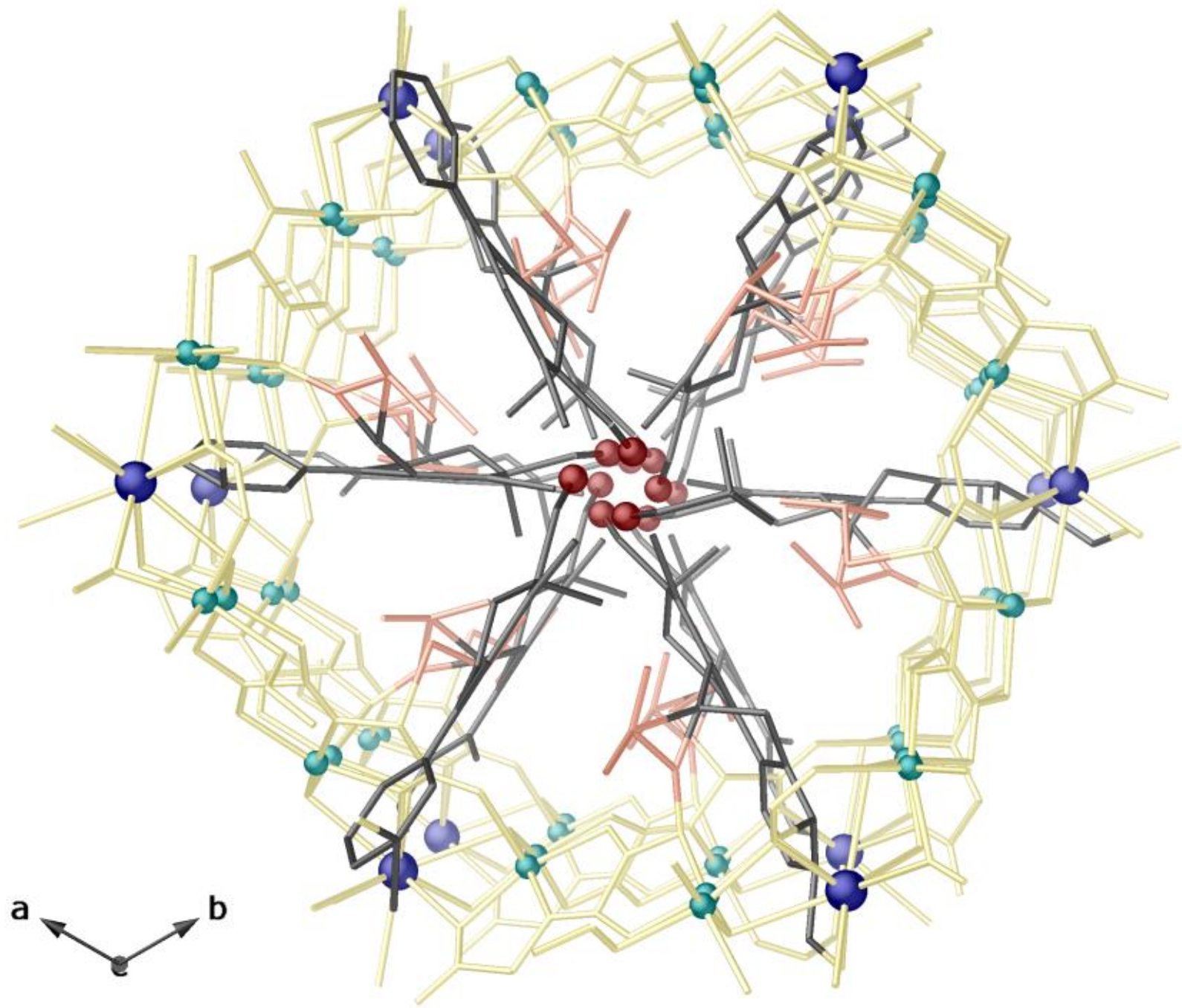

Figure S8. View of a single channel in the crystal structure of **E2@3** evidencing the most stable conformation of 17-β-estradiol guest molecules along the *c* axis. The model highlights the statistical disorder imposed by the space group symmetry: although three symmetry-equivalent positions are generated within the pores, only one is physically occupied, resulting in a 1:3 statistical occupancy. Calcium and copper metal ions are represented by blue and cyan spheres, respectively; organic ligands and guest E2 molecules are represented with gold, gray and red spheres for the oxygen atoms in E2 molecule, respectively.

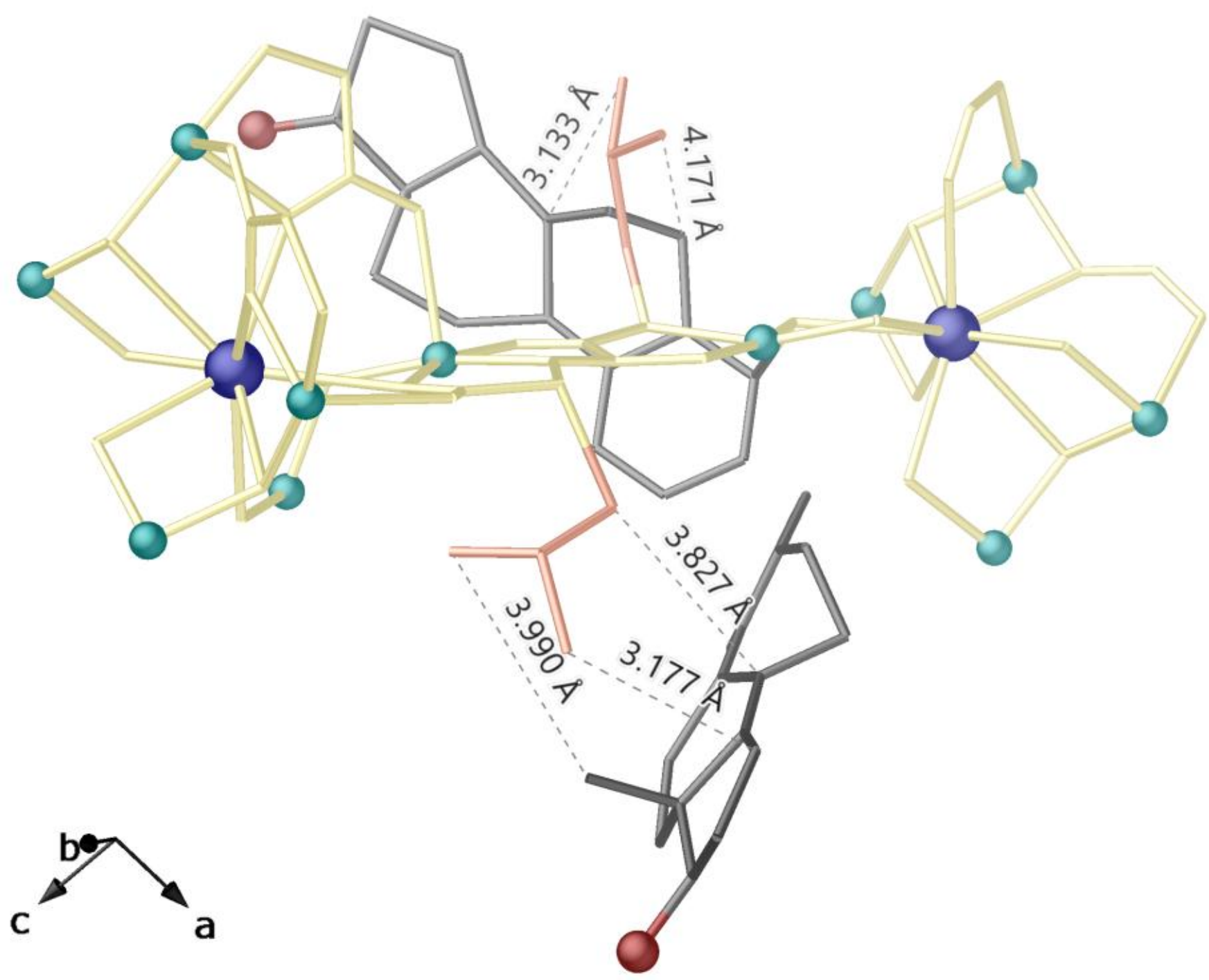

Figure S9. Details of multiple host-guest interactions (dashed lines) in **E2@3** involving leucine residue methyl groups. Calcium and copper metal ions are represented by blue and cyan spheres, respectively; organic ligands and guest E2 molecules are represented with gold, gray and red spheres for the oxygen atoms in the E2 molecule, respectively.

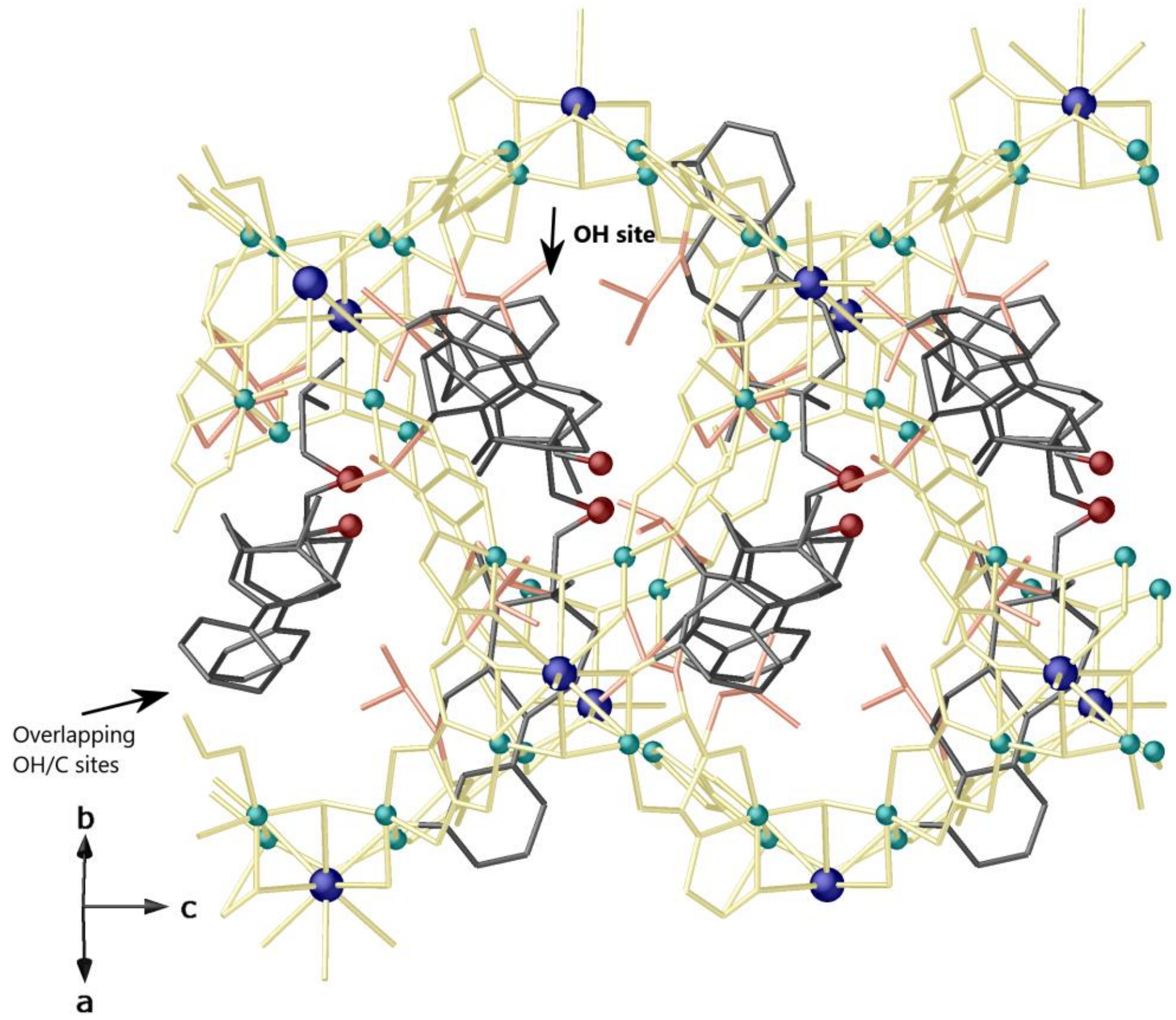

Figure S10. Perspective view of a single channel/pore in the crystal structure of **E2@3** underlying the hydroxyl site is coincident with carbon positions generated by symmetry (not shown for the sake of clarity), reflecting statistical disorder within the structure. Calcium and copper metal ions are represented by blue and cyan spheres, respectively; organic ligands and guest E2 molecules are represented with gold, gray and red spheres for the oxygen atoms in the E2 molecule, respectively.

References

[1] T. Grancha, J. Ferrando-Soria, J. Cano, P. Amorós, B. Seoane, J. Gascon, M. Bazaga-García, E.R. Losilla, A. Cabeza, D. Armentano, E. Pardo, Insights into the Dynamics of Grotthuss Mechanism in a Proton-Conducting Chiral bio MOF, Chem. Mater. 28 (2016) 4608–4615. doi:10.1021/acs.chemmater.6b01286.

[2] M. Mon, F. Lloret, J. Ferrando-Soria, C. Martí-Gastaldo, D. Armentano, E. Pardo, Selective and Efficient Removal of Mercury from Aqueous Media with the Highly Flexible Arms of a BioMOF, Angew. Chem. Int. Ed. 55 (2016) 11167–11172. doi:10.1002/anie.201606015.

[3] M. Mon, J. Ferrando-Soria, T. Grancha, F.R. Fortea-Pérez, J. Gascon, A. Leyva-Pérez, D. Armentano, E. Pardo, Selective Gold Recovery and Catalysis in a Highly Flexible Methionine-Decorated Metal–Organic Framework, J. Am. Chem. Soc. 138 (2016) 7864–7867. doi:10.1021/jacs.6b04635.

[4] T. Grancha, M. Mon, J. Ferrando-Soria, J. Gascon, B. Seoane, E. V. Ramos-Fernandez, D. Armentano, E. Pardo, Tuning the selectivity of light hydrocarbons in natural gas in a family of isoreticular MOFs, J. Mater. Chem. A. 5 (2017) 11032–11039. doi:10.1039/C7TA01179B.

[5] M. Mon, R. Bruno, R. Elliani, A. Tagarelli, X. Qu, S. Chen, J. Ferrando-Soria, D. Armentano, E. Pardo, Lanthanide Discrimination with Hydroxyl-Decorated Flexible Metal–Organic Frameworks, Inorg. Chem. 57 (2018) 13895–13900. doi:10.1021/acs.inorgchem.8b02409.

[6] M. Mon, R. Bruno, E. Tiburcio, M. Viciano-Chumillas, L.H.G. Kalinke, J. Ferrando-Soria, D. Armentano, E. Pardo, Multivariate Metal–Organic Frameworks for the Simultaneous Capture of Organic and Inorganic Contaminants from Water, J. Am. Chem. Soc. 141 (2019) 13601–13609. doi:10.1021/jacs.9b06250.

[7] M. Baratta, T.F. Mastropietro, R. Bruno, A. Tursi, C. Negro, J. Ferrando-Soria, A.I. Mashin, A. Nezhdanov, F.P. Nicoletta, G. De Filpo, E. Pardo, D. Armentano, Multivariate Metal–Organic Framework/Single-Walled Carbon Nanotube Buckypaper for Selective Lead Decontamination, ACS Appl. Nano Mater. 5 (2022) 5223–5233. doi:10.1021/acsanm.2c00280.

[8] C. Negro, H. Martínez Pérez-Cejuela, E.F. Simó-Alfonso, J.M. Herrero-Martínez, R. Bruno, D. Armentano, J. Ferrando-Soria, E. Pardo, Highly Efficient Removal of Neonicotinoid Insecticides by Thioether-Based (Multivariate) Metal–Organic Frameworks, ACS Appl. Mater. Interfaces. 13 (2021) 28424–28432. doi:10.1021/acsami.1c08833.

[9] P. Evans, Scaling and assessment of data quality, Acta Crystallogr. Sect. D Biol. Crystallogr. 62 (2006) 72–82. doi:10.1107/S0907444905036693.

[10] P.R. Evans, G.N. Murshudov, How good are my data and what is the resolution?, Acta Crystallogr. Sect. D Biol. Crystallogr. 69 (2013) 1204–1214. doi:10.1107/S0907444913000061.

[11] M.D. Winn, C.C. Ballard, K.D. Cowtan, E.J. Dodson, P. Emsley, P.R. Evans, R.M. Keegan, E.B. Krissinel, A.G.W. Leslie, A. McCoy, S.J. McNicholas, G.N. Murshudov, N.S. Pannu, E.A. Potterton, H.R. Powell, R.J. Read, A. Vagin, K.S. Wilson, Overview of the CCP 4 suite and current developments, Acta Crystallogr. Sect. D Biol. Crystallogr. 67 (2011) 235–242. doi:10.1107/S0907444910045749.

[12] G. Winter, xia2 : an expert system for macromolecular crystallography data reduction, J. Appl. Crystallogr. 43 (2010) 186–190. doi:10.1107/S0021889809045701.

[13] G. Winter, D.G. Waterman, J.M. Parkhurst, A.S. Brewster, R.J. Gildea, M. Gerstel, L. Fuentes-Montero, M. Vollmar, T. Michels-Clark, I.D. Young, N.K. Sauter, G. Evans, DIALS : implementation and evaluation of a new integration package, Acta Crystallogr. Sect. D Struct. Biol. 74 (2018) 85–97. doi:10.1107/S2059798317017235.

[14] G.M. Sheldrick, A short history of SHELX., Acta Crystallogr. A. 64 (2008) 112–22. doi:10.1107/S0108767307043930.

[15] G.M. Sheldrick, Crystal structure refinement with SHELXL, Acta Crystallogr. Sect. C Struct. Chem. 71 (2015) 3–8. doi:10.1107/S2053229614024218.

[16] W.S.-2013/4 B.A.X.I. Madison, SHELXTL-2013/4, Bruker Analytical X-ray Instruments, Madison, WI, (2013).

[17] A.L. Spek, PLATON SQUEEZE: a tool for the calculation of the disordered solvent contribution to the calculated structure factors, Acta Crystallogr. Sect. C Struct. Chem. 71 (2015) 9–18. doi:10.1107/S2053229614024929.

[18] A.L. Spek, Structure validation in chemical crystallography, Acta Crystallogr. Sect. D Biol. Crystallogr. 65 (2009) 148–155. doi:10.1107/S090744490804362X.

[19] L.J. Farrugia, WinGX suite for small-molecule single-crystal crystallography, J. Appl. Crystallogr. 32 (1999) 837–838. doi:10.1107/S0021889899006020.

[20] C. Palmer, D. CRYSTAL MAKER, Cambridge University Technical Services, CRYSTAL MAKER, Cambridge University Technical Services, Cambridge, (1996).
